# TRPC3 Channel Activity and Viability of Purkinje Neurons can be Regulated by a Local Signalosome

**DOI:** 10.3389/fmolb.2022.818682

**Published:** 2022-02-21

**Authors:** Naveed Aslam, Farah Alvi

**Affiliations:** ^1^ BioSystOmics, Houston, TX, United States; ^2^ Department of Physics, COMSATS University Islamabad, Lahore Campus, Pakistan

**Keywords:** Ca^2+^ influx, channel activation, Ca^2+^ homeostasis, DAG homeostasis, local signalosome

## Abstract

Canonical transient receptor potential channels (TRPC3) may play a pivotal role in the development and viability of dendritic arbor in Purkinje neurons. This is a novel postsynaptic channel for glutamatergic synaptic transmission. In the cerebellum, TRPC3 appears to regulate functions relating to motor coordination in a highly specific manner. Gain of TRPC3 function is linked to significant alterations in the density and connectivity of dendritic arbor in Purkinje neurons. TRPC3 signals downstream of class I metabotropic glutamate receptors (mGluR1). Moreover, diacylglycerol (DAG) can directly bind and activate TRPC3 molecules. Here, we investigate a key question: How can the activity of the TRPC3 channel be regulated in Purkinje neurons? We also explore how mGluR1 activation, Ca^2+^ influx, and DAG homeostasis in Purkinje neurons can be linked to TRPC3 activity modulation. Through systems biology approach, we show that TRPC3 activity can be modulated by a Purkinje cell (PC)–specific local signalosome. The assembly of this signalosome is coordinated by DAG generation after mGluR1 activation. Our results also suggest that purinergic receptor activation leads to the spatial and temporal organization of the TRPC3 signaling module and integration of its key effector molecules such as DAG, PKC*γ*, DGK*γ*, and Ca^2+^ into an organized local signalosome. This signaling machine can regulate the TRPC3 cycling between active, inactive, and desensitized states. Precise activity of the TRPC3 channel is essential for tightly regulating the Ca^2+^ entry into PCs and thus the balance of lipid and Ca^2+^ signaling in Purkinje neurons and hence their viability. Cell-type–specific understanding of mechanisms regulating TRPC3 channel activity could be key in identifying therapeutic targeting opportunities.

## Introduction

The first discovery of a transient potential channel (TRP) was recorded in a *Drosophila* mutant displaying a transient receptor potential in response to steady light signals (Minke 1977; Montell, et al., 1985). Later, the human TRPC genes were discovered (Zhu et al., 1996). Postsynaptic, canonical transient receptor potential channel (TRPC3) is critical for motor coordination (Minke and Cook 2002; Prestori et al., 2020). This channel regulates synaptic transmission through mGluR1-dependent slow EPSCs in cerebellar Purkinje cells (PCs) (Henning, 2011; Shuvaev et al., 2011; Prestori et al., 2020). The TRPC family of channels is widely expressed in the brain, but TRPC3 is the most abundantly expressed subunit in PCs (soma and dendrites) (Hartmann and Konnerth 2009; Tiapko and Groschner 2018). TRPC3 expression increases during postnatal cerebellar development and remains high in the fully developed cerebellum, indicating the possibility of a crucial role during normal motor behavior in mammals (Huang et al., 2007; Nelson and Glitsch 2012; Prestori et al., 2020). Evidence in knockout mice link impaired walking behavior to TRPC3 (Hartmann et al., 2008; Hartmann and Konnerth 2009). Additional observations also show the complete absence of slow synaptic potentials and mGluR1-mediated inward currents in TRPC3 knockout mice, whereas Ca^2+^ release from intracellular stores remains unaffected (Hartmann et al., 2008; Hartmann and Konnerth 2009). Interestingly, observations based on two-photon imaging studies indicate that, despite the high permeability of the channel, TRPC3-mediated Ca^2+^ influx was only 8.8% of the overall mGluR1-mediated Ca^2+^ signal, suggesting only a minor contribution to Ca^2+^ release during physiological conditions (Henning, 2011). However, despite this minor component, TRPC3-mediated Ca^2+^ influx seems to be critical, as behavioral studies show significant impairment of motor control in TRPC3-deficient mice compared with wild types (Hartmann et al., 2008; Hartmann and Konnerth 2009; Henning, 2011).

In the mammalian central nervous system, a functionally critical subfamily of G-protein–coupled receptors (GPCRs) is metabotropic glutamate receptors (mGluRs). The mGluR subfamily consists of eight members, that is, mGluR1 to mGluR8. The receptors of this subfamily are classified into three groups: mGluR1 and mGluR5 belong to group I; mGluR2 and mGluR3 belong to group II; mGluR4, mGluR6, mGluR7 and mGluR8 are group III. Normally, group I receptors are stimulatory, whereas groups II and III receptors are inhibitory. Both mGluR1 and mGluR5 are expressed in PCs. Interestingly, their expression in PCs seems to be mutually exclusive with mGluR5 dominating early in the development and mGluR1 taking over during adult life (Casabona et al., 1997; Notartomaso et al., 2013). This may raise the possibility that during adult life mGluR1 may be acting as a repressor of mGluR5 expression and probably function. Intriguingly, this may mean that persistent elevation of mGluR5 expression during adulthood is probably due to unstable structure in the cerebellum. This is supported by an observation in 30-week-old SCA1 mice showing reduced mGluR1 mRNA and protein levels but enhanced mGluR5 expression levels. This is interesting as usually mGluR5 expression levels are undetectable this late in adult life. Another critical difference between mGluR1 and mGluR5 is the postactivation Ca^2+^ profiles in PCs. The mGluR1 is linked to transient rise, followed by plateau phase, whereas the mGluR5 is characterized by Ca^2+^ oscillations (Baude et al., 1993; Casabona et al., 1997; Nakanishi et al., 1998; Nicoletti et al., 2011; Notartomaso et al., 2013).

Observations in cerebellar PCs suggest that TRPC3 is required for both normal motor behavior and normal synaptic function (Hartmann et al., 2008; Hartmann and Konnerth 2009; Henning, 2011; Tiapko and Groschner 2018). However, the exact role of this channel in modulating Ca^2+^ entry in response to diacylglycerol (DAG), activated PKCγ and Ca^2+^ store depletion, remains controversial. Interestingly, animals carrying gain-of-function (moonwalker mice) (Becker 2014) and loss-of-function (TRPC3 null) mutations (Hartmann et al., 2008; Hartmann and Konnerth 2009; Henning, 2011) have similar phenotypes. It is perplexing as to how one can resolve the ataxia associated with hyper channel activity with impaired motor function associated with loss of activity. It seems that just the precise degree of TRPC3 activity is both necessary and sufficient for normal physiology of PCs. It also appears that, in response to a multitude of factors such as stress, photochromic lipids, kinases, and phosphatases, the TRPC3 channels may cycle between inactive/dormant, active, and desensitized states (Clapham 2003; Tiapko and Groschner 2018). These cycling characteristics may vary with cell types, and tuning the TRPC3 cycling events between these states could provide some therapeutic opportunities (Tiapko and Groschner 2018). Here, we study the mechanisms modulating the TRPC3 cycling between three different states. We propose a hypothesis that a DAG-modulated local signaling module might be able to regulate the interstate cycling of TRPC3 channels in Purkinje neurons. We investigate this hypothesis by proposing a three-compartment model involving Ca^2+^, DAG, PKCγ, DGKγ, and TRPC3 in a signaling loop. The model described here provides a mechanistic basis of TRPC3-mediated Ca^2+^ influx and how the underlying subsignaling network might control the homeostasis of second messenger DAG and calcium influx–mediated Ca^2+^ profiles in membrane and cytosolic compartments in PCs as well as in CHO cells (Fierro et al., 1998).

This work explores how the TRPC3-mediated local signalosome may regulate the gating characteristics of TRPC3 channels in PCs and CHO cells. The following experimental observations may provide some rationality for this investigation: (1) In CHO cells, ATP-induced purinergic receptor activation leads to DAG generation at the membrane, which in turn induces a local functional coupling between its effector molecules, thus ultimately restoring the DAG and Ca^2+^ homeostasis (Yamaguchi et al., 2006; Adachi et al., 2008). (2) In PCs, mutant PKCγ is constitutively active, whereas wild-type PKCγ is inactive, during basal conditions (Shuvaev et al., 2011; Wong et al., 2018). (3) The wild-type PKCγ-mediating gating characteristics of TRPC3 channels are normal. In contrast, mutant PKCγ gating is dysregulated in CHO cells and PCs (Adachi et al., 2008; Shuvaev et al., 2011). (4) DAG directly binds and activates TRPC3 channels (Tiapko and Groschner 2018). (5) PKC modulates the phosphorylation and desensitization of TRPC3 channels in HEK293 cells (Trebak et al., 2005; Venkatachalam et al., 2003: HEK293 cells and DT40 cells). (6) In PCs, the WT PKCγ quickly translocates to the membrane on a KCl-modulated membrane-depolarization event and has a membrane residence time of 18 s (Shuvaev et al., 2011). (7) TRPC3 activation allows Ca^2+^ flux in CHO cells and PCs (Yamaguchi et al., 2006; Adachi et al., 2008; Shuvaev et al., 2011). (8) The Ca^2+^ influx is responsible for PKCγ and DGKγ translocation to the membrane in CHO cells (Yamaguchi et al., 2006; Adachi et al., 2008). (9) Evidence suggests that TRPC3 signals are downstream of mGluR1 (Hartmann et al., 2008). (10) Observations suggest that the mGluR1–PKCγ–TRPC3 pathway may trigger a negative feedback loop, which could be responsible for limited dendritic arborization in Purkinje neurons (Becker et al., 2009).

In this work, we tested the hypothesis that purinergic receptor activation in both cell types (PCs and CHO cells) generates DAG, which in turn controls its own levels through interplay of complex positive and negative feedback loops by integrating Ca^2+^/DAG, PKCγ, DGKγ, and TRPC3 into a subsignaling network. This work tests this hypothesis by constructing a minimal three-compartment TRPC3 model. This model accounts for extracellular Ca^2+^ and its influx into cellular space through membrane-bound TRPC3 molecules. This model suggests that when DAG is generated at the membrane after purinergic receptor activation, it binds and activates the TRPC3 channel, which in turn modulates the entry of extracellular Ca^2+^ into intracellular compartments through hydrophilic pore structures. This cytosolic Ca^2+^ stimulates the translocation of PKCγ and DGKγ from the cytosol to the membrane. Once at the membrane, DAG also activates PKCγ, which in turn phosphorylates and activates DGKγ. The active and phosphorylated DGKγ induces DAG metabolism at the same time. Intriguingly, almost simultaneously, active PKCγ begins to phosphorylate the active TRPC3 channel, thus desensitizing it and reducing the Ca^2+^ influx and hence cytosol-to-membrane migration. This effect of PKCγ-modulated TRPC3 desensitization creates a positive feedback influence on local DAG concentrations in the membrane compartment, as restricting the cytosol-to-membrane translocation of its effector molecules will reduce the local negative feedback leading to its metabolism. In turn, this positive feedback signal on DAG will activate more TRPC3 channels, thus pushing it toward the active state and allowing Ca^2+^ influx. Thus, this model suggests that complex and dynamic interplay of these negative and positive loops could ensure the specificity and robust precision required to maintain TRPC3 signaling during normal physiological conditions in PCs.

## Materials and Methods

### Biochemical Reactions

#### Minimal Model of TRPC3 Signaling in PCs

**FIGURE 1:**
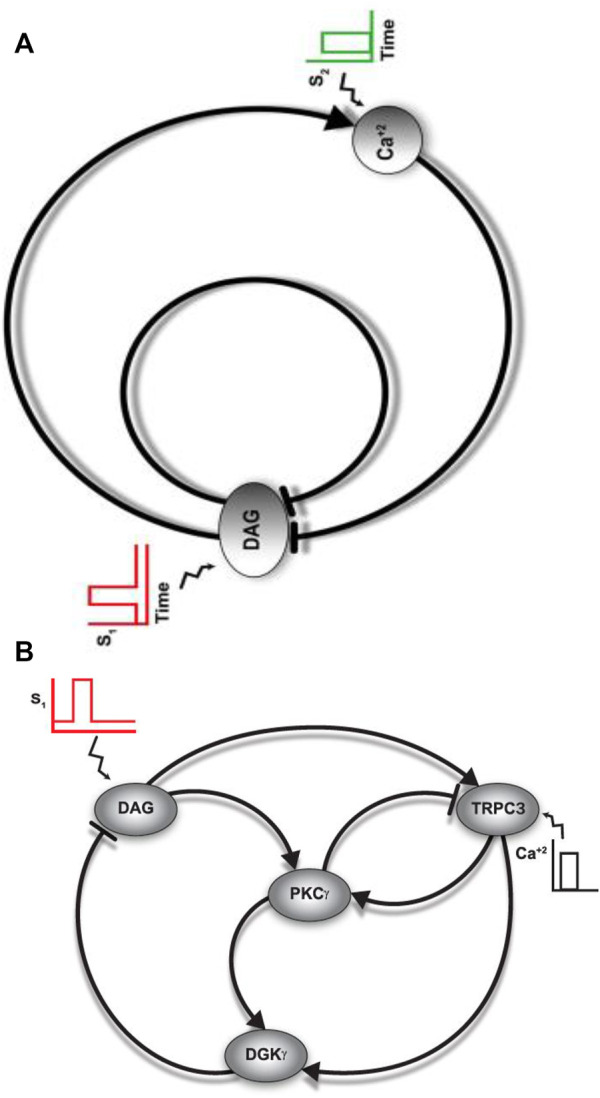
**(A)** Conceptual framework of how TRPC3 signaling might be regulated in PCs. Inception of this subsignaling network in PCs suggests that homeostasis in DAG signaling could be modulated by self-induced, double-negative feedback loops. This highlights the degree of specificity and precision in DAG signaling, which might be required for viability of PCs. **(B)** A detailed wiring diagram of TRPC3 signaling in PCs. This negative feedback effect is generated because of DAG binding and activation of TRPC3 and PKCγ molecules. The direct binding of DAG with TRPC3 leads to its activation. TRPC3 activation leads to calcium influx, thus enhancing the translocation of both PKCγ and DGKγ from cytosol to the plasma membrane. The direct binding of DAG with PKCγ leads to its activation in the membrane compartment. The active PKCγ, in turn, leads to the phosphorylation and activation of DGKγ in the membrane compartment. Thus, the combined effect of translocation and activation leads to negative feedback on DAG as seen by DGKγ-mediated phosphorylation of DAG which induces its metabolism.

**FIGURE 2:**
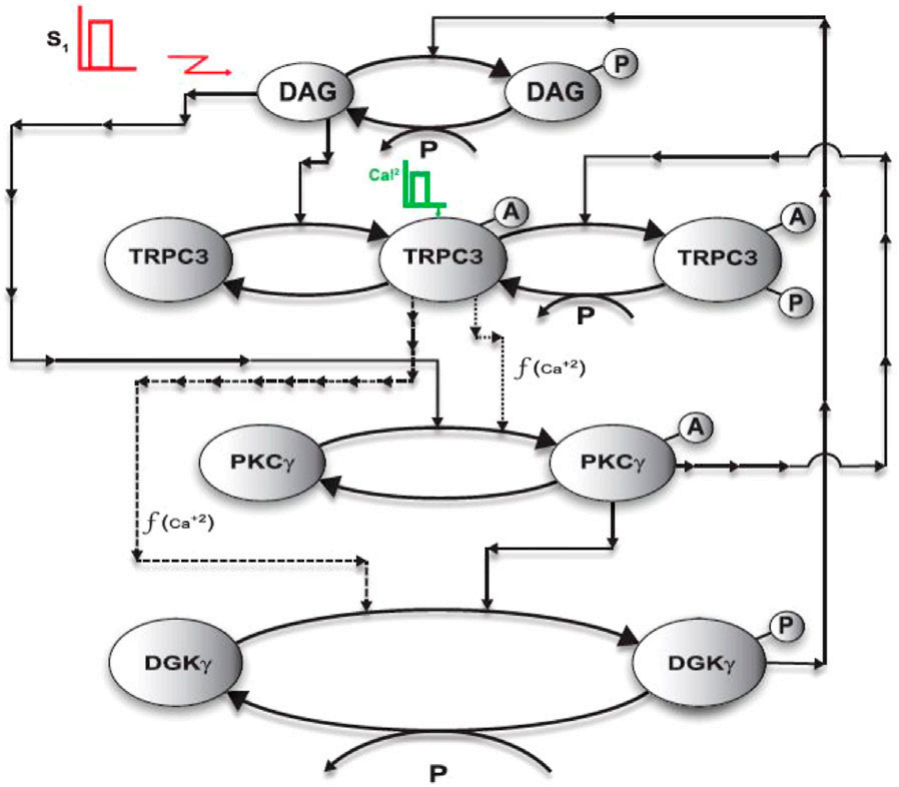
A detailed description of a TRPC3 regulatory loop in PCs. This loop shows that a brief pulse stimulation leads to DAG generation at the membrane. Second messenger DAG, in turn, binds with the TRPC3 channel and PKCγ molecule, thus activating them. The active TRPC3 channel contributes to this regulatory loop by modulating the Ca^2+^ flux from the extracellular to intracellular space, thus inducing the cytosol-to-membrane translocation of both PKCγ and DGKγ (second feedback effect). The active PKCγ leads to the phosphorylation and desensitization of the active TRPC3 channel, as well as phosphorylation and activation DGKγ. The active and phosphorylated DGKγ induces DAG metabolism by phosphorylating it. This loop shows that local generation of DAG in PCs leads to activation of double-negative feedback loops, thus exquisitely regulating the homeostasis in DAG signaling.

**FIGURE 3:**
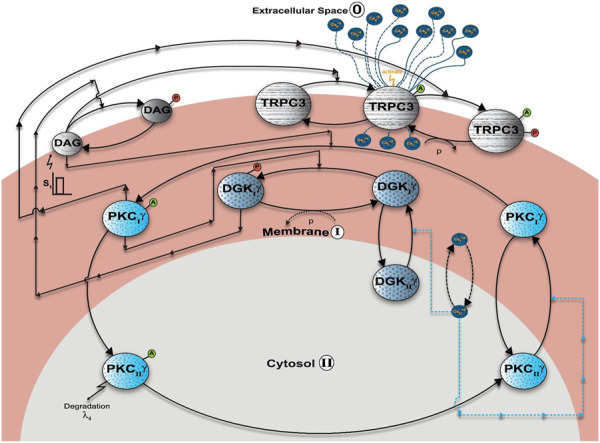
The three-compartment regulatory model of TRPC3 signaling in PCs. This model is based on minimal signaling of TRPC3s in PCs. Here, the first compartment is extracellular space (0), the second compartment is the membrane (I), and the third compartment is cytosol (II). This model explains the spatial and temporal regulation of the DAG–TRPC3–PKCγ–DGKγ axis in PCs. This model describes a sequence of complex events involving the generation of second messengers, translocation, activation, desensitization, and redistribution of DAG axis molecules, that is, TRPC3, PKCγ, and DGKγ. This representation is specific to PCs. Here, the PKCγ molecule can be in four states: (1) dormant PKCγ residing in the cytosol (PKC_II_γ); (2) active PKCγ residing in the cytosol (PKC_II_γ^A^); (3) inactive PKCγ residing in the membrane (PKC_I_γ); and (4) active PKCγ residing in the membrane (PKC_I_γ^A^). The other DAG effector kinase, that is, DGKγ can be in three states: (1) dormant molecule residing in the cytosol (DGK_II_γ); (2) inactive molecule residing in the membrane (DGK_I_γ); and (3) active and phosphorylated molecule residing in the membrane (DGK_I_γ_P_). The DAG effector channel TRPC3 can be in three states: (1) inactive channel residing in the membrane (TRPC3); (2) active channel molecule residing in the membrane (TRPC3^A^); and (3) phosphorylated and desensitized channel molecule residing in the membrane (TRPC3_P_^A^). Here, the channel molecules are located only in the membrane compartment and do not migrate to other compartments. Here, second messenger DAG can either be in nonphosphorylated form (DAG) or in phosphorylated form (DAG_P_). Second messenger calcium can be either in the extracellular compartment (Ca_0_^2+^), membrane compartment (Ca _I_^2+^), or cytosolic compartment (Ca _II_^2+^). This three-compartment model explains that depolarization-induced activation of purinergic receptor leads to DAG generation in the membrane compartment, which in turn binds with channel TRPC3 and activates it, thus inducing calcium influx from the extracellular compartment to intracellular space. This calcium influx stimulates the translocation of both PKC*γ* and DGK*γ* from the cytosolic compartment to the membrane. Once in the membrane compartment, PKC*γ* is activated through DAG binding; this active molecule, in turn, leads to the phosphorylation and activation of DGKγ in the membrane compartment. The active and phosphorylated DGK*γ*, in turn, induces the metabolism of DAG by catalyzing its phosphorylation. The active γ isoform of PKC also leads to negative feedback effect by phosphorylating and desensitizing the TRPC3 channel. This phosphorylation and desensitization event, in turn, leads to a positive feedback effect on the local DAG concentration, as it reduces the calcium influx from the extracellular compartment, thus reducing the cytosol-to-membrane translocation of both DAG effector molecules.

This section describes the biochemical interactions characterizing the local TRPC3 signaling module in PCs. Interactions of this loop are conceptualized in Figures 1–3. These interactions are based on standard Michaelis–Menten–type kinetics. The following sets of biochemical reactions are used to describe the molecular interactions of this loop: the dynamic variables used are Ca^2+^ to describe calcium, DAG to represent the second messenger DAG, DGKγ to represent DAG kinase, PKCγ to represent the γ isoform of protein kinase C, and TRPC3 to represent the canonical transient receptor potential channel. Subscript 0 represents the concentration in the first compartment, that is, extracellular space; subscript I represents the concentration in the second compartment, that is, plasma membrane; and subscript II denotes the concentration in the third compartment, that is, cytosol. Superscript A represents the activated form of the molecule, and subscript P represents the phosphorylated form of the molecule. The phosphatase P is approximated as a fixed parameter. The parameter S1 denotes mGluR1-induced stimulation, leading to the rapid generation of the DAG molecule at the plasma membrane, whereas the parameter S2 denotes Ca^2+^ pulse stimulation in the extracellular space.

$$S_{1}\overset{k_{1}}{\to }DAG$$ (1)

$$S_{2}\overset{k_{27}}{\to }Ca^{2+}0$$ (2)

$$DAG\;+\;TRPC3\overset{k_{13}}{\underset{k_{14}}{⇄}}TRPC3^{A}$$ (3)

$$Ca^{2+}0\overset{k_{15}}{\underset{k_{16}}{⇄}}Ca^{2+}I$$ (4)

$$Ca^{2+}I\overset{k_{17}}{\underset{k_{18}}{⇄}}Ca^{2+}II$$ (5)

$$Ca^{2+}II+PKC_{II}\gamma \overset{k_{19}}{\underset{k_{20}}{⇄}}PKC^{*}II\gamma$$ (6)

$$Ca^{2+}II+DGKII\gamma \overset{k_{21}}{\underset{k_{22}}{⇄}}DGK^{*}II\gamma$$ (7)

$$PKC_{II}\gamma^{*}\overset{\lambda_{0}}{\to }PKC_{I}\gamma$$ (8)

$$DGK_{II}\overset{*}{\gamma }\overset{\lambda_{5}}{\to }DGK_{I}\gamma$$ (9)

$$PKC_{I}\gamma \overset{\lambda_{00}}{\to }PKC_{II}\gamma +Ca^{2+}II$$ (10)

$$DGK_{I}\gamma \overset{\lambda_{55}}{\to }DGK_{II}\gamma +Ca^{2+}II$$ (11)

$$DAG+PKC_{I}\gamma \overset{k_{2}}{\underset{k_{3}}{⇄}}PKC_{I}\gamma^{A}$$ (12)

$$DGK_{I}\gamma +{\left[PKC_{I}\gamma \right]}^{A}\overset{k_{4}}{\underset{k_{5}}{⇄}}\;C_{1}\overset{k_{6}}{\to }{\left[PKC_{I}\gamma \right]}^{A}+DGK_{I}\gamma_{P}$$ (13)

$$DGK_{I}\gamma_{P}+P\overset{k_{7}}{\to }DGK_{I}\gamma \;+P$$ (14)

$$DGK_{I}\gamma_{P}+DAG\overset{k_{8}}{\underset{k_{9}}{⇄}}C_{2}\overset{k_{10}}{\to }DGK_{I}\gamma_{P}+DAG_{P}$$ (15)

$$PKC_{I}\gamma^{A}\overset{\lambda_{3}}{\to }PKC_{II}\gamma^{A}$$ (16)

$$PKC_{II}\gamma^{A}\overset{\lambda_{4}}{\to }\left[\right]$$ (17)

$$PKC_{II}\gamma^{A}\overset{k_{0}}{\to }PKC_{II}\gamma +DAG+Ca^{2+}II$$ (18)

$$DAG_{P}\overset{k_{11}}{\to }P.A$$ (19)

$$DAG_{P}+P\overset{k_{12}}{\to }DAG+P$$ (20)

$${\left[TRPC3\right]}^{A}+{\left[PKC_{I}\gamma \right]}^{A}\overset{k_{23}}{\underset{k_{24}}{⇄}}C_{3}\overset{k_{25}}{\to }{\left[PKC_{I}\gamma \right]}^{A}+{\left[TRPC3\right]}_{P}^{A}$$ (21)

$${\left[TRPC3\right]}_{P}^{A}\;+\;P\overset{k_{26}}{\to }{\left[TRPC3\right]}^{A}+P$$ (22)

#### Full Model of TRPC3 Signaling in PCs

The full model of TRPC3 signaling in PCs is based on above Eqs. 1–22 and following additional biochemical reaction events as described below by Eqs. 23–25.

$${\left[TRPC3\right]}^{A}+\left[VDCC\right]\overset{k_{27}}{\underset{k_{28}}{⇄}}\;C_{4}\overset{k_{29}}{\to }{\left[VDCC\right]}^{A}+{\left[TRPC3\right]}^{A}$$ (23)

$$Ca^{2+}0\overset{k_{30}}{\underset{k_{31}}{⇄}}Ca^{2+}I$$ (24)

$$Ca^{2+\;}\;STORES\overset{k_{32}}{\underset{k_{33}}{⇄}}Ca^{2+}II$$ (25)

The signaling event described by Eq. 23 shows that the active form of TRPC3 channel induces the activation of voltage-dependent Ca^2+^ channels (VDCCs) channel in the membrane compartment. The biochemical event described by Eq. 24 represents the Ca^2+^ from extracellular space into intracellular space due to the activation of VDCC channel. The release of Ca^2+^ from internal stores due to DAG/IP3-mediated activation of store channels is represented by biochemical reaction event as described by Eq. 25.

### Induction

During simulations, membrane depolarization–induced activation of purinergic receptor and DAG generation was simulated by a brief 1.0-min pulse of local biosynthesis of second messenger in the membrane compartment, which induced the translocation and activation of DAG effector molecules.

### Temporal Dynamics

The differential equations resulting from the above interactions, Eqs. 1–22, were integrated through nonlinear solvers using MATLAB (MathWorks). The biochemical rate constants of this system (Eqs. 1–22) are estimated through a systematic process. The first step of this process is calibration of overall model structure. For this purpose, we used data from observations in CHO cells (Yamaguchi et al., 2006; Adachi et al., 2008). The datasets from these observations (Yamaguchi et al., 2006; Adachi et al., 2008) provide the following set of information: (1) translocation intensity of PKCγ and DGKγ from cytosol to membrane in response to ATP-induced purinergic receptor activation; (2) average residence times of PKCγ and DGKγ in the membrane compartment; (3) colocalization time of PKCγ and DGKγ in the membrane compartment; (4) retranslocation kinetics of both these molecules back to cytosol; (5) kinetics of DAG generation and resolution in the membrane after stimulation; (6) kinetics of P.A. generation in the membrane; (7) Ca^2+^ elevation and resolution profiles; (8) TRPC3 activation kinetics by DAG; and (9) TRPC3 deactivation characteristics by active PKCγ in the membrane compartment. With this information in hand, we tested several topological structures of this signaling module and selected the structure proposed here in Figures 1–3. We selected this structure because with this we are able to match above data from CHO cells by estimating, testing, and selecting various combinations of biochemical rate constants. Our final choice is reported herein (Supplementary Material S2: Supplementary Table S1 **)**. This choice is based on the best available information. After this calibration phase of model structure, we used this signaling topology to estimate the biochemical rate constants of the above system in PCs. The relevant observations in PCs (Shuvaev et al., 2011) provide the following set of information: (1) kinetics of PKCγ translocation; (2) intensity of PKCγ translocation; (3) average residence time of PKCγ in the membrane compartment; and (4) TRPC3 deactivation properties by active PKCγ in the membrane compartment. Unfortunately, we have no direct information available on the DGKγ translocation properties in PCs, we therefore assumed that DGKγ translocation patterns in PCs will follow the same mechanisms as observed in CHO cells (Yamaguchi et al., 2006), although with different kinetics, that is, the kinetics matching to PKCγ translocation in PCs. The PKCγ translocation kinetics is much faster in PCs (average residence time of 19 s) compared with PKCγ translocation kinetics in CHO cells (average residence time of 1.8 min). With this information in hand, we designed several combinations of parameter set values representing PC dynamics. Our best selection of parameters matching closely to PC observations is reported herein (Supplementary Material S3: Supplementary Table S2). Unless otherwise stated, all molecular concentrations in the model were expressed as picograms per milliliter, and time was represented in seconds.

## Results

### Three-Compartment Regulatory Model of the TRPC3 and Ca^2+^-Assisted DAG Signaling Cascade

The minimal local regulatory model (Figures 1–3) we proposed for TRPC3 channel regulation in PCs is composed of five components: (1) second messenger Ca^2+^, which can be in three states, extracellular Ca^2+^_0_, membrane Ca^2+^_I_, or cytosolic Ca^2+^_II_; (2) second messenger DAG, which can be in two states, nonphosphorylated at membrane or phosphorylated at membrane; (3) PKC_I_γ, which can be in one of four states, cytosolic dormant PKC_II_γ, inactive membrane PKC_I_γ, active membrane PKC_I_γ^A^, or active cytosolic PKC_II_γ^A^; (4) DGKγ, which can be in one of three states, cytosolic (DGK_II_γ), inactive membrane (DGK_I_γ), or active phosphorylated membrane (DGK_I_γ_P_); and (5) TRPC3 channel molecule, which can be in one of three states, membrane inactive (TRPC3), membrane active (TRPC3^A^), or membrane active phosphorylated (TRPC3^A^_P_). Here, two independent second messenger pulse stimulations are used to perturb this model. One pulse stimulation mimics the generation of DAG in response to membrane depolarization–induced purinergic receptor activation, and a second pulse represents the elevation of Ca^2+^ levels in the extracellular space above the basal levels. This model suggests that membrane depolarization–induced purinergic receptor activation generates DAG in the membrane compartment, which in turn binds with the inactive TRPC3 channel in the membrane compartment and activates it. The activation of TRPC3 channel induces Ca^2+^ flux from extracellular space to intracellular region, thus stimulating the migration of DAG effector molecules from cytosol to membrane. The sensitivity of translocation varies as PKCγ responds to a nanomolar rise in Ca^2+^ levels in the cytosol, whereas DGKγ requires a micromolar increase in Ca^2+^ levels for its migration toward membrane (Yamaguchi et al., 2006; Adachi et al., 2008). Once PKCγ is in the membrane compartment, DAG binds and activates it. This active molecule, in turn, leads to the phosphorylation and activation of DGKγ in the membrane compartment. The active and phosphorylated DGKγ in the membrane induces the metabolism of second messenger DAG by phosphorylating it. This model suggests that dual action of DAG on the TRPC3 channel and PKCγ ensures tight regulation of DAG levels (self-limiting signaling cascade, supported by negative feedback) and maintenance of its homeostasis in PCs during post–purinergic receptor activation mode.

Further details of the four PKCγ states and three DGKγ states can be found in our previous modeling work based on CHO and PCs (Aslam and Alvi, 2017; Aslam and Alvi, 2020; Aslam and Alva 2019). However, unlike our previous work, here we assume that the translocation of PKCγ and DGKγ from the cytosol to the membrane is regulated through kinetic events that are described by proportionality functions of the Ca^2+^ concentration (which is more consistent with previous experimental observations in CHO cells (Yamaguchi et al., 2006). We also assume nonnegligible basal levels of dormant PKCγ (10 pg/mL), DGKγ (10 pg/mL), and TRPC3 (4 pg/mL), whereas the basal concentrations of all other forms of PKCγ, DGKγ, DAG, and Ca^2+^ were considered negligibly insignificant. For the biochemical reaction and translocation rates, we used data from CHO cells and PCs (Yamaguchi et al., 2006; Adachi et al., 2008; Shuvaev et al., 2011). This, model suggests that TRPC3 channel cycling events between inactive, active, and active-phosphorylated but desensitized states support the self-limiting cascade of DAG signaling. This type of mechanism ensures that rate of local DAG generation is counterbalanced by DGKγ-assisted rate of DAG removal. Thus, under normal conditions, this is how the exquisite regulation of the DAG levels in the plasma membrane can be managed. Interestingly, this model also suggests that active PKCγ molecules can phosphorylate and desensitize the TRPC3 channel in the membrane compartment, which in turn will reduce Ca^2+^ flux from extracellular into intracellular space, thus reducing the translocations of both PKCγ and DGKγ from the cytosol to the membrane. According to this model, PKCγ-assisted TRPC3 phosphorylation will create a positive feedback effect on local DAG levels, as the translocation of effector molecules from cytosol to plasma membrane is reduced. This might suggest that altered TRPC3 gating characteristics can be linked to prolonged DAG accumulation at the membrane. Although the proposed model is a simplified representation of ATP-assisted TRPC3 gating in CHO cells and membrane depolarization–induced TRPC3 gating characteristics in PCs, we structured this model to capture essential features of the DAG–TRPC3–Ca^2+^–PKCγ–DGKγ signaling axis. This local DAG–TRPC3 cascade is described by a set of biochemical reactions (Eqs. 1–22). In both the cell systems, local biosynthesis of DAG (as mimicked here by application of a brief pulse) precedes certain molecular events such as the activation of phospholipase C (PLC) and PLC-mediated hydrolysis of phosphatidylinositol 4,5-biphosphate to produce inositol triphosphate (IP3) and DAG. These events are not accounted, here as we are only focusing on downstream signaling of the purinergic receptor pathway. We modeled the local biosynthesis of DAG by using a brief pulse. This is used to mimic the effects of ATP and KCl on purinergic receptors in CHOs and PCs. This is a simpler approach, and it ignores many details of active purinergic receptor-mediated biosynthesis of DAG; therefore, we chose this simple representation as not to obscure our model with details.

In order to study the effects of Ca^2+^ release and additional influx by VDCC channels on the TRPC3 signaling, we also constructed a full TRPC3 signalosome model (Supplementary Figure S1). This full TRPC3 signaling model is composed of six components: (1) second messenger Ca^2+^, which can be in four states: extracellular Ca^2+^_0_, membrane Ca^2+^_I_, cytosolic Ca^2+^_II_, internal stores Ca^2+^_Stores_; (2) second messenger DAG, which can be in two states: nonphosphorylated at membrane or phosphorylated at membrane; (3) PKC_I_γ, which can be in one of four states: cytosolic dormant PKC_II_γ, inactive membrane PKC_I_γ, active membrane PKC_I_γ^A^, or active cytosolic PKC_II_γ^A^; (4) DGKγ, which can be in one of three states: cytosolic (DGK_II_γ), inactive membrane (DGK_I_γ), or active phosphorylated membrane (DGK_I_γ_P_); (5) TRPC3 channel molecule, which can be in one of three states: membrane inactive (TRPC3), membrane active (TRPC3^A^), or membrane active phosphorylated (TRPC3^A^_P_); and (6) VDCC channel molecule, which can be in two states: inactive channel residing in membrane (VDCC) or active molecule residing in the membrane (VDCC^A^) (Hashimotoa et al., 2011; Henning, 2011). This version of TRPC3 model is also perturbed by two simultaneous second messenger pulse stimulations. One pulse stimulation mimics the generation of DAG and IP3 in response to membrane depolarization–induced purinergic receptor activation, and a second pulse represents the elevation of Ca^2+^ levels in the extracellular space above the basal levels. This model suggests that on membrane depolarization–induced purinergic receptor activation, DAG is generated in the membrane compartment, which in turn binds with the inactive TRPC3 channel at the membrane and activates it. This model also suggests that purinergic receptor activation also leads to IP3 generation, which in turn diffuses to cytosol and induces the Ca^2+^ release from intracellular stores. For the sake of simplicity, it is assumed here that IP3 biosynthesis and degradation responses are analogous to DAG, so for practical purpose, the rate constants describing Ca^2+^ release events are developed as a function of DAG. In addition, it is also assumed here that TRPC3 channel may permeate monovalent cations and can depolarize local dendrites, which may activate VDCCs and induce Ca^2+^ influx (Hashimotoa et al., 2011; Henning, 2011). The VDCC channel is activated by the active form of TRPC3. Here, the channel molecules are located only in the membrane compartment and do not migrate to other compartments. The additional events of full model are described by biochemical reaction events (Eqs 23–25).

### Signaling Characteristics of the TRPC3-Mediated Signalosome in PCs and CHO Cells

**FIGURE 4:**
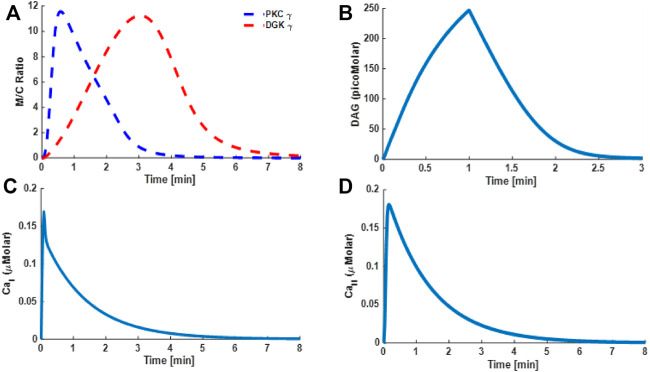
The simulations based on a minimal model and mimicking the TRPC3 signaling characteristics. These results are based on the data from CHO cells. These results are based on store depletion version of model and without VDCCs. Thus, only Ca^2+^ contribution here is coming from TRPC3-mediated influx. These results show the translocation characteristics of the PKCγ and DGKγ molecular pair and dynamics of second messenger DAG and Ca^2+^ during ATP-induced purinergic receptor activation and stimulation of the TRPC3 signaling cascade. These results also show that, in response to a brief (1 min) pulse, DAG is generated in the plasma membrane leading to Ca^2+^ influx and rapid translocation of PKCγ and DGKγ from cytosol to membrane. The translocation of these molecules is measured by the membrane-to-cytosol (M/C) ratio. These results suggest that as soon as DAG is generated at the plasma membrane in response to pulse “S_1_,” it activates the TRPC3 channel, thus inducing the calcium flux from extracellular space into membrane compartment, which in turn induces the translocation of first PKC*γ* (due to higher Ca^2+^ sensitivity), followed by DGK*γ* (due to lower Ca^2+^ sensitivity). Once at the membrane, PKC*γ* is activated by binding with DAG. The active PKC*γ* phosphorylates and activates DGK*γ* and phosphorylates TRPC3, thus destabilizing it. The active and phosphorylated DGKγ, in turn, leads to DAG metabolism, thus reducing the DAG levels, and as DAG levels drop to baseline, both these effector molecules return to the cytosol. **(A)** M/C ratio of PKCγ and DGKγ; **(B)** DAG temporal dynamics; **(C)** Ca^2+^ temporal dynamics in membrane compartment; **(D)** Ca^2+^ temporal dynamics in cytosolic compartment.

**FIGURE 5:**
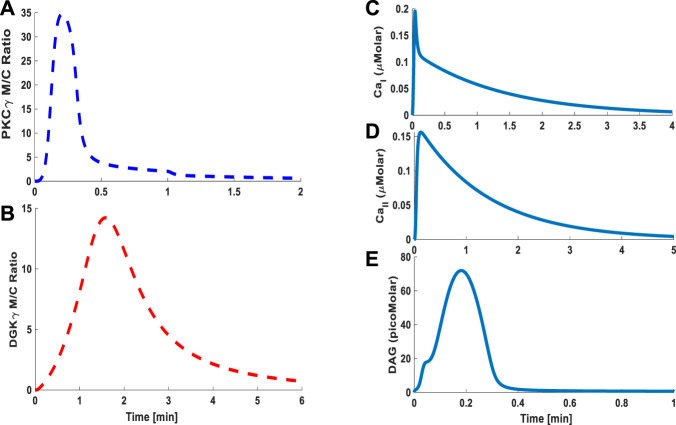
Simulations based on a minimal model mimicking the TRPC3 signaling characteristics. These results are based on the data from Purkinje cells. These results are based on store depletion version of model and without VDCCs. Thus, only Ca^2+^ contribution here is coming from TRPC3-mediated influx. These results show the translocation characteristics of the PKCγ and DGKγ molecular pair and dynamics of second messenger DAG and Ca^2+^ during KCl-induced purinergic receptor activation and stimulation of the TRPC3 signaling cascade in PCs. These results show that, in response to a brief 1-min pulse, DAG is generated at the membrane, thus activating the TRPC3 channel, which in turn allows the Ca^2+^ flux into the intracellular space and stimulates the translocation of PKCγ and DGKγ from cytosol to membrane. The translocation of these molecules is measured by the membrane-to-cytosol (M/C) ratio. The intensity and kinetics of PKCγ translocation in response to purinergic receptor activation as measured by M/C ratio of PKCγ and DGKγ. **(A)** M/C ratio of PKCγ and DGKγ; **(C)** DAG temporal dynamics; **(C)** Ca^2+^ temporal dynamics in membrane compartment, **(D)** Ca^2+^ temporal dynamics in cytosolic compartment; **(E)** DAG temporal dynamics.

Through our three-compartment minimal model, we next determined the signaling characteristics of the DAG–TRPC3 subsignaling network in CHO and PCs. The signaling characteristics were determined by measuring the M/C ratio of both DAG effector molecules, that is, PKCγ and DGKγ, and temporal dynamics of Ca^2+^ and DAG. The results reported here are based on slower kinetics in CHO cells (Figure 4) and faster kinetics in PCs (Figure 5) (Yamaguchi et al., 2006; Adachi et al., 2008; Shuvaev et al., 2011). Membrane-to-cytosol (M/C) translocation characteristics of both DAG effector molecules, that is, PKCγ and DGKγ, are a key component of this subsignaling loop. These characteristics are determined by measuring the speed of migration from the cytosol to the membrane, membrane residence time/colocalization time, and intensity of translocation (M/C ratio) of both the DAG effector molecules. The M/C ratios of PKCγ and DGKγ in this model are also an indirect index of their activation and describe the relative distributions of PKCγ and DGKγ in membrane and cytosolic compartments. A higher M/C ratio suggests high migration tendencies from the cytosol to the membrane. For the results based on mimicking the CHO cell migration tendencies, we approximated ATP-induced activation of GPCR and subsequent DAG generation through a brief 1-min pulse. The strength of pulse is described by an arbitrary parameter S_1_; herein, the parameter S_1_ is set at the arbitrary level of 7. In the absence of pulse, there is no *de novo* DAG biosynthesis, as the system is fixed at a basal state with both molecules in the cytosol and no possibility of translocation. In the simulations mimicking the CHO cells, the application of pulse leads to DAG generation at the membrane (Figure 4B), which in turn activates membrane-bound TRPC3, thus allowing calcium flux from extracellular compartments to intracellular compartments of the membrane and cytosol, resulting in the elevation of Ca^2+^ concentrations in these compartments (Figure 4C and D, Ca^2+^_I_ and Ca^2+^_II_). Elevated Ca^2+^ in the cytosol stimulates cytosol-to-membrane migration of PKCγ and DGKγ, and the M/C ratio of both molecules increased to a maximum level followed by gradual clearance (Figure 4).

During the migration process, the temporal evolution of PKCγ and DGKγ indicates how these molecules shuttled between compartments. The temporal dynamics are depicted in Figure 4 and clearly show two phases of TRPC3-mediated translocation kinetics: an early Ca^2+^ influx-driven phase, in which both these molecules migrate to the membrane in response to DAG generation and subsequent TRPC3 activation, followed by a resolution phase in which negative feedback effects prevail, thus reducing the Ca^2+^ influx through TRPC3 inactivation and resulting in remigration of both these molecules to the cytosol to their dormant forms. These results show that *de novo* DAG synthesis and TRPC3-mediated Ca^2+^ influx are critical for molecular migration of both the DAG effector molecules in this signaling cascade. Interestingly, simulations mimicking the PCs showed similar characteristics, but the kinetics are much faster (Figure 5) (Shuvaev et al., 2011). These results show that, on membrane depolarization–induced activation of purinergic receptor, DAG is generated at the membrane (Figure 5E), leading to TRPC3 activation, calcium influx, and elevation of calcium levels in the membrane (Figure 5C) and cytosolic (Figure 5D) compartments. This results in quick migration of PKCγ from the cytosol to the membrane (Figure 5A) followed by DGKγ migration (Figure 5B). Once at the membrane, negative feedback is generated, resulting in loss of TRPC3 activation and remigration of both these DAG effector molecules from the membrane to the cytosol.

**FIGURE 6:**
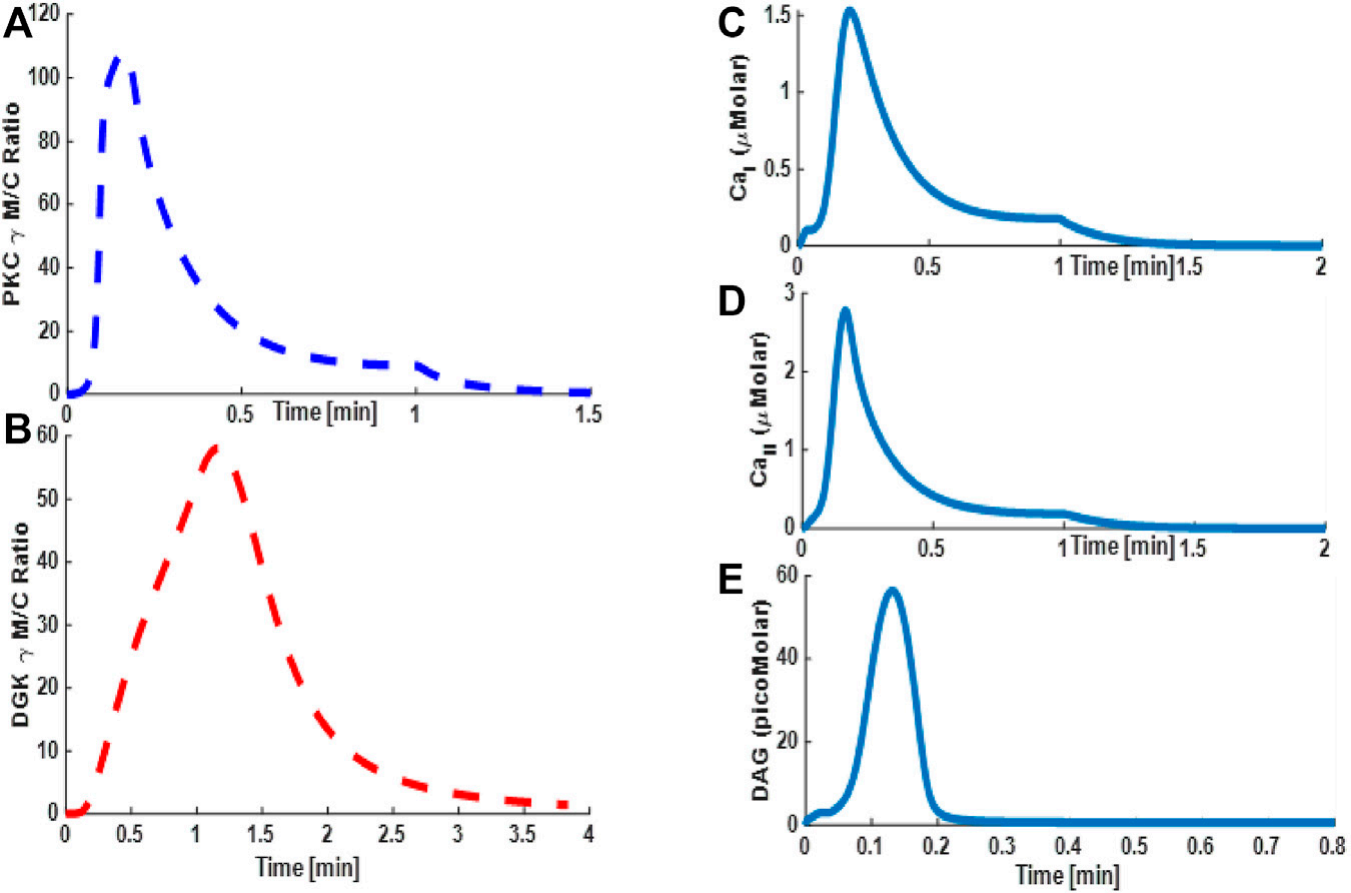
Simulations based on a full model and mimicking the TRPC3 signaling characteristics based on the data from Purkinje cells. These results are based on the full model of TRPC3 signaling, which along with TRPC3-mediated influx also accounts for cytosolic release of Ca^2+^ from stores and VDCC-mediated influx in the membrane compartment. Thus, Ca^2+^ release and flux contributions are as follows: (1) release from internal stores; (2) TRPC3-mediated influx; and (3) VDCC-mediated influx. These results show the translocation characteristics of the PKCγ and DGKγ molecular pair and dynamics of second messenger DAG and Ca^2+^ during KCl-induced purinergic receptor activation and stimulation of the TRPC3 signaling cascade in PCs. These results show that, in response to a brief 1-min pulse, DAG is generated at the membrane, thus activating TRPC3 channel, which in turn allows the calcium flux into intracellular space and stimulates the translocation of PKCγ and DGKγ from cytosol to membrane. **(A)** M/C ratio of PKCγ; **(B)** M/C ratio of DGKγ; **(C)** Ca^2+^ temporal dynamics in membrane compartment; **(D)** Ca^2+^ temporal dynamics in cytosolic compartment; **(E)** DAG temporal dynamics.

Next, we compared the signaling properties of full TRPC3 model in PCs (Figure 6) with minimal model (Figure 5). Results from the full model (Figure 6) show much higher-intensity Ca^2+^ response signals both in the membrane (Figure 6C) and cytosolic (Figure 6D) compartments compared with the minimal model case (Figures 5C,D). These results also reveal that in the simulations representing full model translocation intensity of both PKCγ and DGKγ is three to four times higher (Figures 6A,B) compared with the minimal model case (Figures 5A,B). Interestingly, the residence time of PKCγ in the membrane compartment is almost the same for both the models (Figure 5A, Figure 6A), thus indicating that although the Ca^2+^ signal is much stronger in full model leading to higher intensity of translocation of key TRPC3 signaling molecules, that is, PKCγ and DGKγ, the kinetics of translocation in both models are almost identical, leading to similar patterns of membrane residence of PKCγ molecule.

### How the DGKγ Activation Rate May Influence TRPC3-Mediated Signaling in CHO Cells and PCs

**FIGURE 7:**
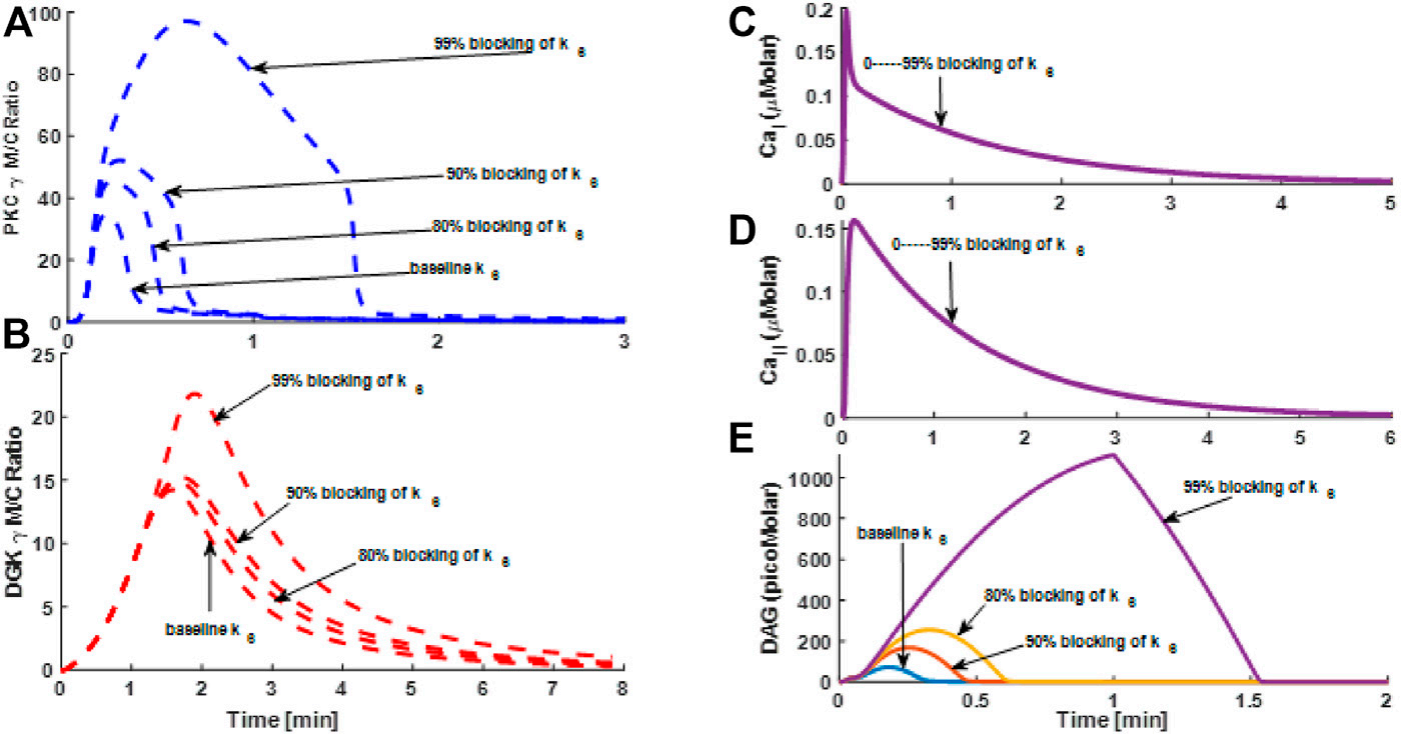
The simulations mimicking the effect of (rate constant describing the phosphorylation of DGK_I_ *γ* by PKC_I_ *γ* at the membrane) “k_6_” blocking on the TRPC3 signaling characteristics based on the data from Purkinje cells. These results are based on store depletion version of model and without VDCCs. Thus, only Ca^2+^ contribution here is coming from TRPC3-mediated influx. These results show the translocation characteristics of PKCγ and DGKγ molecular pair and dynamics of second messenger DAG and Ca^2+^ during KCl-induced purinergic receptor activation and stimulation of the TRPC3 signaling cascade in PCs. These results show that, in response to a brief 1-min pulse, DAG is generated at the membrane, thus activating TRPC3 channel, which in turn allows the calcium flux into intracellular space and stimulates the translocation of PKC*γ* and DGK*γ* from cytosol to membrane. **(A)** M/C ratio of PKC*γ* at different blocking levels of k_6_; **(B)** M/C ratio of DGKγ at different blocking levels of k_6_; **(C)** Ca^2+^ temporal dynamics in membrane compartment; **(D)** Ca^2+^ temporal dynamics in cytosolic compartment; **(E)** DAG temporal dynamics.

DGKγ phosphorylation and activation at the membrane could play a crucial role in TRPC3-mediated signaling both in CHO cells and PCs. As this key event determines the degree of DAG’s positive influence on TRPC3 and PKCγ, it in turn regulates Ca^2+^ influx and the overall negative feedback effect on second messengers and thus their homeostasis. We next set out to address the following question: How might perturbing the DGKγ activation rate influence the signaling properties of PKCγ and DGKγ in the TRPC3-modulated subsignaling network? We selected the phosphorylation rate of DGKγ by active PKCγ, “k_6_” at the membrane as a perturbing parameter. First, we used minimal model to study the effects of k_6_ on TRPC3 signaling in both PCs and CHOs (Figure 7 and Supplementary Figure S2). In our simulations mimicking the PCs, we compared four different cases: (1) baseline k_6_; (2) 80% blocking of k_6_; (3) 90% blocking of k_6_; and (4) 99% blocking of k_6_ (Figure 7). We also compared two different cases in CHO cells but with different blocking levels: (1) no blocking or baseline k_6_ and (2) 99.99% k_6_ blocking (Supplementary Material SI: Supplementary Figure S2). Our results showed that blocking parameter “k_6_” led to an increase in the maximum M/C ratio as well as average residence time of PKCγ in PCs (Figure 7A). Our results also showed that in PCs the τ_PKCγ_ was almost doubled from no blocking case to 90% blocking (Figure 7A: τ_PKCγ_ was 18 s at no blocking and 38 s at 90% blocking). Our results also showed that blocking “k_6_” increased the maximum M/C ratio of DGKγ, but the average residence time of DGKγ only slightly increased with an increase in k_6_ blocking. Additional simulations based on CHO cells also showed that τ_PKCγ_ increased from 1.8 to 2.6 min at 99.99% blocking (Supplementary Material SI: Supplementary Figure S2). However, there was an interesting difference across cell types, as in the case of CHO cells, 0% to 99.99% blocking only led to a 24% increase in τ_PKCγ_, whereas 0% to 90% blocking in PCs was linked to almost a 100% increase in τ_PKCγ_.

Next, we also studied how blocking the parameter, “k_6_” in full model of TRPC3 might influence the signaling (Supplementary Figure S8). Our results showed that blocking, “k_6_” led to increase in M/C ratio, as well as increase in the membrane residence time of PKCγ (Supplementary Material SI: Supplementary Figure S8A). Our simulations also showed that blocking k_6_ in full model led to significant increase in the M/C ratio of DGKγ (Supplementary Material SI: Supplementary Figure S8B), which contrasts with increase in the minimal model for PCs (Figure7B) but decrease in CHO cells (Supplementary Material SI: Supplementary Figure S2A). Interestingly, the k_6_ blocking simulations based on the minimal model for both PCs (Figures 7C,D) and CHOs (Supplementary Material SI: Supplementary Figures S2C,D) were not showing any effect on Ca^2+^ signals (compared with baseline case); however, for the case of k_6_ blocking in full model, the strength and persistency duration of Ca^2+^ signal were increased (Supplementary Material SI: Supplementary Figures S8C,D).

### Simulations Mimicking the Effect of TRPC3 Expression on Signaling Properties of PKCγ and DGKγ and Second Messengers DAG and Ca^2+^ in PCs and CHO Cells

**FIGURE 8:**
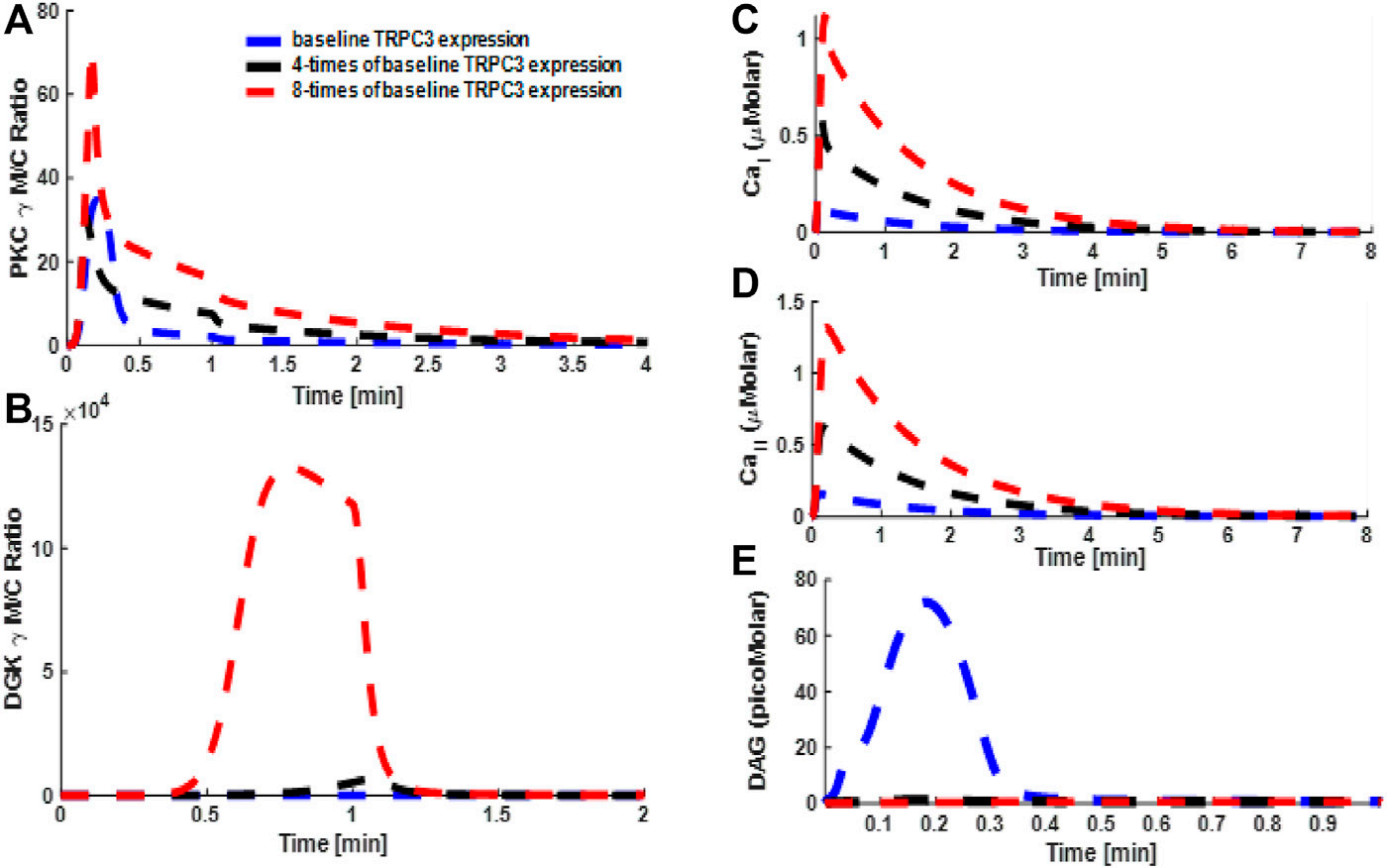
Simulations mimicking the TRPC3 signaling characteristics based on the data from Purkinje cells. These results are based on the full model of TRPC3 signaling, which along with TRPC3-mediated influx also accounts for cytosolic release of Ca^2+^ from stores and VDCC-mediated influx in the membrane compartment. Thus, Ca^2+^ release and flux contributions are as follows: (1) release from internal stores; (2) TRPC3-mediated influx; and (3) VDCC-mediated influx. These results show the translocation characteristics of the PKCγ and DGKγ molecular pair and dynamics of second messenger DAG and Ca^2+^ during KCl-induced purinergic receptor activation and stimulation of the TRPC3 signaling cascade in PCs. These results show that, in response to a brief 1-min pulse, DAG is generated at the membrane, thus activating TRPC3 channel, which in turn allows the calcium flux into intracellular space and stimulates the translocation of PKCγ and DGKγ from cytosol to membrane. **(A)** M/C ratio of PKCγ; **(B)** M/C ratio of DGKγ; **(C)** Ca^2+^ temporal dynamics in membrane compartment; **(D)** Ca^2+^ temporal dynamics in cytosolic compartment; **(E)** DAG temporal dynamics.

The convoluted interplay of negative and positive feedback loops proposed in this study might also critically depend on the expression levels of the TRPC3 channel both in CHO cells and PCs. Through a minimal model, we next determined how perturbing TRPC3 expression might influence the signaling properties of the PKCγ–DGKγ molecular pair and temporal dynamics of second messengers in both these cell types (Figure 8 [PCs] and Supplementary Material SI: Supplementary Figure S4 [CHOs]). For simulations, mimicking the PCs, we selected three different expression levels of TRPC3 for PCs: (1) baseline, (2) four times of baseline, and (3) eight times of baseline (Figure 8). In addition, for simulations mimicking CHO cells, two different expression levels (Supplementary Material SI; Supplementary Figure S4) were selected: (1) baseline TRPC3 expression and (2) two times of baseline expression. Here, we applied a brief pulse for 1 min, which led to rapid DAG generation at the plasma membrane. Our results based on minimal PC model showed that increasing the TRPC3 expression in PCs led to an increase in the maximum M/C ratio of PKCγ, whereas the residence time of PKCγ in the membrane compartment was almost the same (Figure 8A). Interestingly, our results also showed that enhancing TRPC3 expression reduced not only the DAG levels, but the duration for which they were nonnegligible was also reduced in the plasma membrane (Figure 8E). Conversely, the Ca^2+^ concentrations and duration for their nonnegligible levels in membrane and cytosolic compartments increased (Figures 8C,D). Interestingly, our results also showed that enhancing the TRPC3 expression led to significant increase in the translocation intensity of DGKγ (Figure 8B). Our additional minimal model simulations of this system based on the data from CHO cells showed similar trends in PKCγ and DGKγ translocation properties (Supplementary Material SI: Supplementary Figure S4). The temporal dynamics of second messengers in CHO cells (Supplementary Material SI: Supplementary Figure S4) also showed similar trends. These results suggest that enhancing TRPC3 expression effectively enhances the influence of negative feedback on second messenger DAG in both cell types, although it will also increase the positive feedback effect (generated by active PKCγ through desensitizing TRPC3), but its influence on enhancing the overall negative effect was more pronounced than the increase in positive feedback effect.

**FIGURE 9:**
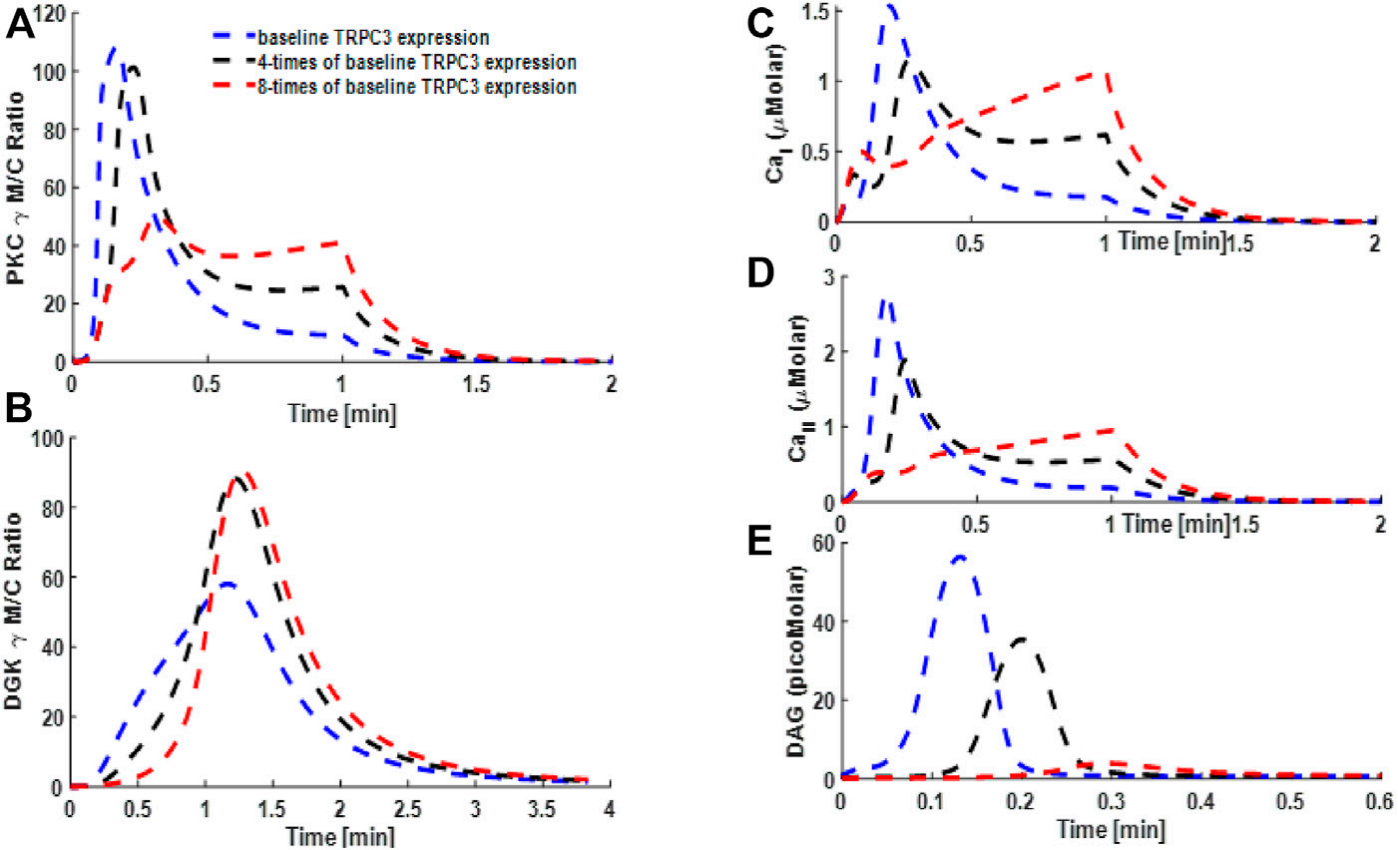
Simulations mimicking the effect of altering the TRPC3 expression on the TRPC3 signaling characteristics based on the data from Purkinje cells. These results are based on the full model of TRPC3 signaling, which along with TRPC3-mediated influx also accounts for cytosolic release of Ca^2+^ from stores and VDCC-mediated influx in the membrane compartment. Thus, Ca^2+^ release and flux contributions are as follows: (1) release from internal stores; (2) TRPC3-mediated influx; and (3) VDCC-mediated influx. These results show the translocation characteristics of the PKCγ and DGKγ molecular pair and dynamics of second messenger DAG and Ca^2+^ during KCl-induced purinergic receptor activation and stimulation of the TRPC3 signaling cascade in PCs. These results show that, in response to a brief 1-min pulse, DAG is generated at the membrane, thus activating TRPC3 channel, which in turn allows the calcium flux into intracellular space and stimulates the translocation of PKCγ and DGKγ from cytosol to membrane. **(A)** M/C ratio of PKCγ; **(B)** M/C ratio of DGKγ; **(C)** Ca^2+^ temporal dynamics in membrane compartment; **(D)** Ca^2+^ temporal dynamics in cytosolic compartment; **(E)** DAG temporal dynamics.

Intriguingly, our simulations based on a full TRPC3 model (Figure 9) showed that enhancing the TRPC3 expression to four and eight times levels in this model reduced the M/C ratio of PKCγ but increased the membrane residence time of this molecule (Figure 9A). Results from full model also showed that translocation intensity, that is, M/C ratio of DGKγ, was increased with increase in the expression levels of TRPC3 (Figure 9B). The Ca^2+^ signals in membrane and cytosol compartments were reduced with rather slower rise to the maximum levels (Figures 9C,D), whereas the intensity and duration of persistency for DAG signals were significantly reduced (Figure 9E).

### How Might the TRPC3 Channel Activation Rate (k_13_) Influence the Signaling Properties of PKCγ–DGKγ and Second Messengers?

The signaling cascade proposed here can be characterized by a robust, negative, self-restricting influence, which second messenger DAG generates on itself to ensure tight regulation of its levels in the membrane compartment after purinergic receptor activation. In the minimal model, this negative influence is critically dependent on DAG’s ability to activate the TRPC3 channel, as without this channel activity, there will be no cytosol-to-membrane translocation of DAG effector molecules and hence no negative feedback (at least as assumed in the minimal model as Ca^2+^ release from intracellular stores is not accounted in the minimal model, and only Ca^2+^ influx contributes to translocation). We next determined how blocking the channel activation rate, “k_13_,” might affect the translocation and signaling properties of different components involved in the TRPC3 cascade (Supplementary Material SI: Supplementary Figure S5). Our results showed that blocking k_13_ in PCs delayed the PKCγs membrane translocation and reduced maximum M/C ratio, but it increased the average residence time of PKCγ in the membrane (Supplementary Materials I: Figure S_5_-a: τ_PKCγ_ = 18 s no blocking case, but 99% blocking led to 42 s). A similar trend was observed with DGKγ signaling (Supplementary Material SI: Supplementary Figure S5B). Intriguingly, these results also showed that blocking k_13_ effectively increased the levels and duration of a nonnegligible concentration of DAG, indicating that negative feedback weakened with channel blocking (Supplementary Material SI: Supplementary Figure S5E). On the other hand, blocking reduced Ca^2+^ levels in both membrane and cytosolic compartments (Supplementary Material SI: Supplementary Figures S5C,D).

Intriguingly, when we developed the k_13_ blocking simulations with full TRPC3 model (Supplementary Material SI: Supplementary Figure S7), our results showed that even for 90% and 99% blocking levels of k_13_, the translocation intensity and membrane residence time of PKCγ almost remained the same as the baseline case (Supplementary Material SI: Supplementary Figure S7A). Although additional results showed that with k_13_ blocking, the intensity of DGKγ translocation was reduced (Supplementary Material SI: Supplementary Figure S7B), but the membrane residence time remained the same as for baseline case. Even more interesting, the blocking of k_13_ did not result in major influence not only on the Ca^2+^signals in cytosol and membrane Supplementary Material SI: Supplementary Figures S7C,D) but also on the overall DAG response (Supplementary Material SI: Supplementary Figure S7E).

### Effect of the PKCγ-Mediated Channel Phosphorylation Rate (k_25_) on the Signaling Properties of the TRPC3 Signaling Cascade

Next, we asked the question how the rate of PKCγ-modulated phosphorylation/desensitization of TRPC3 channels could influence the signaling properties of this local signaling module. Based on the minimal model, our results showed that by increasing the phosphorylation/desensitization rate, “k_25_” reduced the PKCγ membrane translocation intensity but increased its membrane residence time (Supplementary Material SI: Supplementary Figure S6A). Our results also showed a similar trend for DGKγ translocation properties in this cascade (Supplementary Material SI: Supplementary Figure S6B). Additional results showed that enhancing k_25_ increased not only the DAG levels but also the duration for which they were nonnegligible (Supplementary Material SI: Supplementary Figure S6E). However, Ca^2+^ levels in both membrane and cytosolic compartments decreased (Supplementary Material SI: Supplementary Figures S6C,D), showing weakening of the negative feedback effect due to channel desensitization and reduction in Ca^2+^ influx to intracellular compartments. Interestingly, in CHO cells, enhancing the k_25_ showed similar behavior (Supplementary Material SI: Supplementary Figure S3).

## Discussion

The cerebellum, an essential part of mammalian brain hierarchy, is critical in regulating higher-level functions such as motor execution, coordination, and learning (Chen et al., 1995; Brkanac et al., 2002; Carlson et al., 2009; Tanaka 2009; Shimobayashi, 2016). PCs play a unique role in cerebellar circuitry as they are the only component that extends outside the cerebellum, so they are crucial in transmitting critical information relating to motor function (Shimobayashi, 2016; Tanaka 2009). From climbing fiber and parallel fiber, these cells receive two main excitatory inputs. Synapses formed at the parallel fiber–PCs interface are characterized by glutamate release from presynaptic parallel fiber stimulation. This generates two postsynaptic responses in PCs: (1) AMPA receptor–modulated fast EPSCs; (2) TRPC3-mediated slow EPSCs (Henning 2011; Shimobayashi, 2016). In the cerebellum, TRPC3 channels are predominantly expressed in PCs (Henning 2011; Hartmann and Kunnerth 2015; Prestori et al., 2020). Interestingly, although the TRPC3 channels regulate approximately only 9% of Ca^2+^ release in PCs, they play a critical role in motor function, as revealed in PCs of moonwalker mice carrying a mutation in the gene encoding TRPC3 channels (Shimobayashi, 2016; Tanaka 2009; Becker 2014; Henning 2011). This moonwalker-related TRPC3 mutation is linked to excessive Ca^2+^ influx, thus leading to dysfunction of PC dendritic development (Becker 2014). Significant differences and impairment in PC dendritic growth and arborization are observed in moonwalker mice compared with TRPC3 KO mice. Additional evidence also suggests that mutation of PKCγ in SCA14 is unable to completely phosphorylate TRPC3 and precisely modulate its gating (Adachi et al., 2008; Sheave et al., 2011). This impairment in channel gating leads to an increase in TRPC3-mediated Ca^2+^ influx and increased amplitude of mGluR1-mediated slow EPSCs. In turn, this may be linked to SCA14-associated neurodegeneration (Duenas et al., 2006; Henning 2011; Shimobayashi, 2016).

During development in rats, as the density of the PC dendritic tree increases, so does the TRPC3 expression (Huang et al., 2007). This is also the only subunit of the TRPC family whose expression increases during development. These high expression levels are maintained during adulthood, suggesting a critical role for this channel in the adult cerebellum function. TRPC3 channels are critical not only for dendritic development (embryogenesis) but also for the viability of PCs (Becker, 2017). In a single PC, the TRPC3 channels are by far the most abundant molecule compared with other members of this family. Observations link the pathological mechanisms underlying several different genetic forms of cerebellar ataxia to TRPC3-orchestrated dysfunction in Ca^2+^ signaling (Konur and Ghosh, 2005; Paulson, 2009; Soong and Paulson, 2007; Kim 2013; Becker et al., 2011; Dunleva et al., 2015; Fogel et al., 2015; Fogel et al., 2016; Prestori et al., 2020). For example, the gene encoding TRPC3 channel proteins may seem to be a critical regulator in the mouse models of SCA1 (Lin et al., 2000; Ingram et al., 2016), SCA2 (Pflieger et al., 2017), and SCA14 (Adachi et al., 2008; Hartmann et al., 2008). A gain in TRPC3 function is believed to be linked to Ca^2+^-dependent degradation of PCs in SCA14 disease (Hartmann et al., 2008; Adachi et al., 2008; Verbeek et al., 2005; Verbeek, et al., 2008; Yamashita, et al., 2000; Yabe, et al., 2003). However, it is not clear how exactly the activity of the TRPC3 channel can be regulated in PCs. This study investigates the precision mechanisms involved in modulating TRPC3 activity in PCs. Here, through a systems biology approach, we develop a model representing a local TRPC3-orchestrated signaling cascade in PCs, which might be responsible for regulating the precision in TRPC3 gating during normal physiological functions of the cerebellum.

Experimental evidence suggests that purinergic receptor activation in PCs and CHO cells can lead to local (membrane compartment) DAG generation, which in turn induces the assembly of a signaling module at the membrane compartment involving PKCγ, DGKγ, and TRPC3 (Glitsch, 2010; Goto and Kondo, 1999; Guzman and Gerevich, 2016; Schrenk, et al., 2002). This local signaling machine becomes functional through DAG-modulated activation of the TRPC3 channel, thus inducing Ca^2+^ influx that in part leads to translocation of PKCγ and DGKγ to the membrane. Once at the membrane, DAG binds with PKCγ and activates DGKγ, which in turn phosphorylates DAG, thus inducing its metabolism. Active PKCγ can also phosphorylate and deactivate TRPC3, thus terminating the Ca^2+^ influx and reducing the cytosol-to-membrane translocation of PKCγ and DGKγ, which in turn will provide positive feedback on the DAG levels in the membrane compartment. Intriguingly, a set of observations in mouse cerebellar Purkinje neurons shows that mGluR1 may physically interact with the TRPC3 signalosome, thereby ensuring the specificity and efficacy of synaptic transmission (Nishizuka, 1988; Saito et al., 1988; Nishizuka, 1992; Nishizuka, 1995; Metzger and Kapfhammer, 2000; Lopez-Bendito et al., 2001; Newton, 2001; Saito and Shirai, 2002; Newton, 2003; Mérida et al., 2008; Kato et al., 2012; Ady et al., 2014). The local cascade based on temporal and spatial integration of DAG–TRPC3–Ca^2+^–PKCγ–DGKγ molecules into a signaling platform provides a robust basis to exquisitely regulate TRPC3 gating, thus ensuring the viability of PCs by precisely regulating Ca^2+^ influx into PCs. Here, we constructed a minimal three-compartment model to study this local signaling module. Our model was based on the extracellular compartment, membrane compartment, and cytosolic compartment. This model enabled us to account for Ca^2+^- and DAG-induced assembly and activation of this signaling machine, thus specifically modulating the temporal as well as spatial signaling characteristics. Moreover, we also developed a full model of TRPC3 signaling in PCs to compare the scenarios of internal Ca^2+^ store depletion (minimal model) with Ca^2+^ release in the cytosol.

The molecular interactions and basic assumptions of this study are based on experimental observations, as described in the above sections. One critical assumption in our three-compartmental model is based on the ability of PKCγ to phosphorylate and deactivate TRPC3 channels. This might be deemed as a controversial assumption, as some observations support but others do not directly confirm this assumption (Venkatachalam et al., 2003; Trebak et al., 2005; Adachi et al., 2008; Nelson and Glitsch 2012). Interestingly, one set of observations suggests that the PKC enzyme could possibly regulate TRPC3 in expression and other native systems, but a contrasting set of observations in rat cerebellar PCs indicates that PKC is unable to modulate the activity of TRPC3 (Venkatachalam et al., 2003; Trebak et al., 2005; Adachi et al., 2008; Nelson and Glitsch 2012). This discrepancy raises an interesting question: Why is PKC not able to phosphorylate TRPC3 channels? Some plausible explanations are listed as follows. (1) It is not completely clear that TRPC3 in rat PCs function as homomeric or heteromeric, as slow EPSCs both in rat and mouse were not completely inhibited by 10 μM Pyr3 (TRPC3 blocker). This partial block suggests that channels underlying slow EPSCs might not be TRPC3 homomeric but rather a cluster of TRPC family members containing TRPC3. Interestingly, in TRPC3 knockout mice, slow EPSCs were eliminated, thus suggesting that TRPC3 might be a direct and only link to slow EPSCs. As in the expression system TRPC3 was overexpressed and was phosphorylated and inhibited by PKC and/or PKG, it is possible that a heterogeneous TRPC family cluster is not subject to phosphorylation and inhibition in native systems. (2) Another possible explanation could be the specific microenvironment, such as lipid rafts, which might restrict the access of PKCγ to phosphorylate and inhibit TRPC3 channels in rat PCs, that is, lack of opportunity for PKCγ in rat PCs rather than lack of ability. Interestingly, another set of observations (Adachi et al., 2008) in CHO, HEK-293, and human neuroblastoma SH-5Y5Y cells indicates that PKCγ can regulate Ca^2+^ influx through modulating the plasma membrane TRPC3 channels. Intriguingly, another observation shows that mGluR1 activation in PCs causes the opening of TRPC3, which is balanced by inhibitory action of PKCγ on TRPC3 channels in PCs, and this phenomenon can be recorded electrophysiologically as slow EPSCs (Shuvaev et al., 2011). Recordings based on PCs show that the amplitude of slow EPSCs significantly increased for mutant PKCγ for moderate and high stimulation cases (Shuvaev et al., 2011). This might suggest that a mutant molecule cancels the inhibitory action of endogenous PKCγ and alters the gating of TRPC3 channels. Yet another set of observations based on mutagenesis and *in vivo* phosphorylation assays on full-length TRPC3 shows that a conserved serine residue at position 712 (S712) seems to be necessary and sufficient for PKC-modulated channel regulation. Even a single-site alanine mutant (S712A) failed to show any *in vivo* phosphorylation or inhibition of channel function (Trebak et al., 2005; Kwan et al., 2006). Based on these critical explanations, it is possible that PKC-modulated phosphorylation and inhibition of TRPC3 channels are regulated differently in different cell types or animals, as the microenvironment that TRPC3 resides in may vary. Thus, we cannot rule out the possibility that PKC is not able to inhibit TRPC3 in PCs; however, further experiments and analyses are needed to resolve differences between native rat PCs and expression systems.

Another critical assumption of this study is regarding the description of cytosol-to-membrane translocation of PKCγ and DGKγ. Here, we have developed and incorporated Ca^2+^-dependent quantitative functions to describe the membrane-directed translocation events (Supplementary Material S2: Supplementary Table S1; Supplementary Material S3: Supplementary Table S2; Supplementary Material S4: Supplementary Table S3). We have also used different sensitivities to describe the translocation rates of PKCγ and DGKγ. Here, the PKCγ translocation event was more sensitive to an increase in Ca^2+^ concentration in the cytosol compared with DGKγ molecule (Supplementary Material S2: Supplementary Table S1; Supplementary Material S3: Supplementary Table S2; Supplementary Material S4: Supplementary Table S3). This is based on previous experimental observations in CHO cells, showing that a nanomolar increase in Ca^2+^ concentration in cytosol is enough to stimulate the translocation of PKCγ, whereas DGKγ translocation requires a micromolar increase in the Ca^2+^ concentration in the cytosol (Yamaguchi et al., 2006). These observations are captured in the current model and described as the slope of linear functions representing the translocation rate of PKCγ and DGKγ. However, there is one limitation to this description, as the Ca^2+^ concentration in cytosol in the minimal model increased only due to TRPC3-modulated Ca^2+^ influx from the extracellular space. This could be questioned, as in reality mGluR1-mediated activation of PCs will involve both Ca^2+^ influx from the extracellular space and Ca^2+^ release from intracellular stores. Here, we assume that Ca^2+^ influx is the only signal and only contributing factor for PKCγ and DGKγ translocation (for the minimal model of TRPC3 signalosome). This is a similar approach in which it is assumed that internal Ca^2+^ stores are being depleted before stimulating the TRPC3 channel, thus using the method to study the signaling effects only caused by TRPC3-modulated influx (Adachi et al., 2008). In reality, this is only 9% of the overall Ca^2+^ signal observed in mGluR1-mediated activation. Certainly, this is a questionable assumption, but here it is incorporated to keep the model simple and relevant to TRPC3-mediated effects on local signaling. Interestingly, although TRPC3-mediated Ca^2+^ influx contributes to only 9% of the overall Ca^2+^ signal, data from previous observations show that blocking this influx leads to major issues in the viability and functionality of PCs, and blocking this influx may lead to dysfunction of PCs leading to abnormal gating and potential neurodegeneration.

Intriguingly, as TRPC3 channel manages only 9% of overall Ca^2+^ signal in PCs, we also asked the question of how the signaling properties of the TRPC3 signalosome might alter if Ca^2+^ release from internal stores and influx from VDCCs are also incorporated in the basic model (Hashimotoa et al., 2011; Henning, 2011). For this, we compared the minimal model (Figure 3), which only accounts for TRPC3-mediated Ca^2+^ influx with the full model (Supplementary Figure S1). Our results show that in contrast to the minimal model, the apex of the curve for Ca^2+^_I_ and Ca^2+^_II_ has shifted to 1.5 and 2.8 micromolar compared with 0.2 and 0.15 micromolar in the minimal model (Figures 5, 6). This demonstrates the effects of Ca^2+^ release from the stores and how it might alter the signaling characteristics of key players in the local signalosome of TRPC3 channels in PCs.

The proposed model of the TRPC3 signaling cascade in this study is based on the data from CHO cells (Figure 4 and Supplementary Material SI: Supplementary Figures S2–4) and PCs (Figures 5–9 and Supplementary Material SI: Supplementary Figures S5–S8). One might wonder why we used two different cell systems for this study. The main reason for doing so is to properly calibrate the model structure and kinetics describing TRPC3 signaling. The datasets coming from CHO cells are more in-depth and are more insightful compared with datasets available from PCs (Yamaguchi et al., 2006; Adachi et al., 2008; Shuvaev et al., 2011). Therefore, first, we synthesized the TRPC3 signaling network based on datasets from CHO cells. Next, we perturbed this network by mimicking ATP-induced stimulation of CHO cells and matched Ca^2+^-induced translocation profiles of PKCγ and DGKγ with available data from CHO cells (Yamaguchi et al., 2006; Adachi et al., 2008). Once the structural and kinetic model calibration was established, we also developed a case for PCs and matched the experimental observations of PKCγs membrane residence time in PCs with our simulations of the TRPC3 signaling module (Shuvaev et al., 2011). This way, we have a robust topological structure of the signaling network and more physiologically relevant biochemical kinetic parameters (Supplementary Material S2: Supplementary Table S1; Supplementary Material S3: Supplementary Table S2) for local TRPC3 channel signaling. Next, we used this information to generate predictions both in CHO cells (Supplementary Material SI: Supplementary Figures S2–4) and in PCs (Figures 7–9 and Supplementary Material SI: Supplementary Figures S5–8).

This study reveals the mechanistic function of TRPC3 channels in the Purkinje neuron physiology. Cycling of TRPC3 between activated, inactivated, and desensitized states may provide specific therapeutic opportunities to target this channel in a precise spatiotemporal manner. Development of pharmacological interventions designed to target TRPC3 channel molecules may open possibilities to modulate the physiology of PCs. Here, through our approach, we investigated some of these possibilities. For example, first, we asked the question of how signaling may alter if we perturb the DGKγ activation rate in this subsignaling module (Figure 7 and Supplementary Material SI: Supplementary Figure S2). Our results showed that blocking the DGKγ activation rate reduced the negative feedback influence, thus enhancing PKCγ signaling in the membrane and its membrane residence time (Figure 7A and Supplementary Material SI: Supplementary Figure: S2A). This is interesting because it means active and membrane localized PKCγ will be able to exert more influence on the TRPC3 channel through desensitizing it, which will effectively reduce or eliminate the Ca^2+^ influx, but despite the reduced Ca^2+^ influx, PKCγ is retained longer at the membrane because of reduced DAG metabolism. Next, we investigated the question of how altering the expression of the TRPC3 channel (Figures 8,9 and Supplementary Material SI: Supplementary Figure S4) may influence the signaling properties of this module. We used minimal model to evaluate the effects of TRPC3 channel expression on local signaling in PCs (Figure 8) and CHOs (Supplementary Material SI: Supplementary Figure S4). We also compared the results from minimal model in CHO and PCs (Figure 8 and Supplementary Material SI: Supplementary Figure S4) with full model in PCs (Figure 9). Our results based on the minimal model in PCs showed that enhancing the expression of the TRPC3 channel not only increased the amplitude of PKCγ membrane translocation (Figure 8A) but also decreased DAG levels (Figure 8E) and increased Ca^2+^ concentrations in membrane and cytosolic compartments (Figures 8C,D). This is possible because enhanced TRPC3 expression in the membrane will stimulate more Ca^2+^ influx into cellular compartments, thus enhancing the PKCγ translocation response. Interestingly, however, the membrane residence time of PKCγ was only slightly reduced (Figure 8A), which might explain the decrease in amplitude and duration of the DAG response (Figure 8E), as only a minor reduction in the membrane residence duration of PKCγ but much higher translocation response will enhance the negative feedback effects on DAG due to enhanced translocation response of DGKγ (Figure 8B) due to increased expression of TRPC3 channel in the membrane compartment. Similar patterns were also observed in the minimal model simulations mimicking the CHO cells (Supplementary Material SI: Supplementary Figure: S4). Interestingly, simulations based on the full model of PCs showed that enhancing TRPC3 expression reduced the membrane translocation of PKCγ (Figure 9A) but enhanced its membrane residence time, whereas the translocation response of DGKγ (Figure 9B) was increased. The DAG signal was significantly reduced (Figure 9E), whereas Ca^2+^ signals were moderately reduced compared with baseline case (Figures 9C,D). Intriguingly, if channel activity is blocked during the simulations mimicking enhanced TRPC3 expression (Supplementary Material SI: Supplementary Figure S9), the signaling characteristics of this local signalosome at least partially seem to be restored.

One might wonder whether there is a rationale for one of the predictions described here, linking changes in TRPC3 expressions (Figures 8, 9 and Supplementary Material SI: Supplementary Figure: S4–S9) to alterations in the M/C ratio and membrane residence time of PKCγ and DGKγ. It is true that there is not a lot of evidence available to support this prediction, but the following observations may provide some rationales: (1) based on the data from cerebellar slices from adult mice, it shows that in TRPC3^−/−^ mice the sEPSCs are totally absent, whereas in mice lacking TRPC1 the sEPSCs are unaffected (Henning, 2011); (2) the amplitude of sEPSCs can be linked to the translocation intensity and membrane residence time of PKCγ in PCs (Shuvaev et al., 2011); (3) in TRPC3-deficient mice, which are also store depleted, the Ca^2+^ influx into cytosol is eliminated and also the possibility of PKCγ and DGKγ translocation (Henning, 2011); (4) mGluR1 activation in PCs stimulates the TRPC3 opening, which can be recorded as sEPSCs (Henning, 2011); (5) the Ca^2+^ imaging in PCs from wild-type and TRPC3-deficient mice show two distinct Ca^2+^ signaling events: Ca^2+^ release from stores and TRPC3-mediated Ca^2+^ influx (Henning, 2011); (6) additional observations in COS-7 cells show that TRPC3 is a substrate of PKCγ, and only wild-type PKCγ can negatively regulate TRPC3 activity but not SCA14 mutant PKCγ because of difference in membrane residence duration (Adachi et al., 2008).

In yet another example based on the minimal model of TRPC3 channel, the blocking activation rate of TRPC3 by DAG binding (Supplementary Material SI: Supplementary Figure: S5) reduced the amplitude of PKCγ membrane translocation but increased its membrane residence time (Supplementary Material SI: Supplementary Figure S5A). These results also showed that blocking TRPC3 activation enhanced the amplitude and duration of the DAG response (Supplementary Material SI: Supplementary Figure S5E) but reduced the Ca^2+^ concentrations and duration of their nonnegligible concentrations in membrane and cytosolic compartments (Supplementary Material SI: Supplementary Figures S5,6). In addition, blocking k_13_ also reduced the translocation intensity of DGKγ but enhanced its membrane residence time (Supplementary Material SI: Supplementary Figure S5B). Interestingly, when we used the full model to study the effects of k_13_ blocking (Supplementary Material SI: Supplementary Figure S7), we found that translocation intensity and the membrane residence time of PKCγ were almost unaffected (Supplementary Material SI: Supplementary Figure S7A). However, the translocation intensity of DGKγ was reduced, but its membrane residence time remained almost the same as baseline case of no blocking (Supplementary Material SI: Supplementary Figure S7B). In addition, these results showed that blocking reduced the DAG levels (Supplementary Material SI: Supplementary Figure S7E). Moreover, the Ca^2+^ signals in membrane and cytosol were also reduced but not to a significant degree (Supplementary Material SI: Supplementary Figures S7C,D), especially when comparing with Ca^2+^ signal reduction in the minimal model (Supplementary Material SI: Supplementary Figures S5C,D).

Last, but not least, we also analyzed how PKCγ-modulated TRPC3 phosphorylation might influence the signaling of this cascade in CHO cells (Supplementary Material SI: Supplementary Figure S3) and PCs (Supplementary Material SI: Supplementary Figure S6). Interestingly, enhancing the TRPC3 desensitization rate significantly reduced the amplitude of PKCγ and DGKγ signaling in the membrane compartment but increased their membrane compartment residence time in both CHO cells (Supplementary Material SI: Supplementary Figure S3A) and PCs (Supplementary Material SI: Supplementary Figure S6A,B). Surprisingly, enhancing the TRPC3 desensitization rate enhanced the amplitude and duration of DAG at the membrane (Supplementary Material SI: Supplementary Figure S3B; Supplementary Material SI: Supplementary Figure S6E) and reduced the Ca^2+^ concentration and temporal duration in the membrane and cytosol (Supplementary Material SI: Supplementary Figure S3C,D; Supplementary Material SI: Supplementary Figures S6C,D). These results suggest that enhancing the desensitization will effectively reduce the negative feedback on DAG, allowing it to persist in the membrane compartment for a longer period (Supplementary Material SI: Supplementary Figure S3B; Supplementary Material SI: Supplementary Figure S6E). Parameters for the full model are given in the supplementary materials (Supplementary Material S4: Supplementary Table S3).

In the TRPC family of channels, the TRPC3 is the only member that is constitutively active and may even lead to increase in local Ca^2+^ levels prior to full channel activation by DAG. Previous observations link the constitutive TRPC3 activity to neuronal excitability (Neuner et al., 2015; Wu et al., 2019; Um et al., 2021). Here, we also incorporated constitutive TRPC3 activity into the minimal model of signalosome (Supplementary Material SI: Supplementary Figure S10). Interestingly, our results show that with constitutively active TRPC3 channel, although the membrane translocation intensity of PKCγ is almost the same as the baseline case of inactive TRPC3 in basal state (Supplementary Material SI: Supplementary Figure S10A **)**, its membrane residence time is slightly reduced, which could be due to enhanced translocation of DGKγ (Supplementary Material SI: Supplementary Figure S10B) and increase in Ca^2+^ influx but reduced signaling of DAG (Supplementary Material SI: Supplementary Figures S10-C–S10-E).

This investigation provides the first quantitative description of a local TRPC3 signalosome in PCs. Although this model is based on supporting observations in PCs, CHOs, HEK 293, DT40, and COS-7 cells, it does not account for all the complexities involving TRPC3, DAG, and Ca^2+^ signaling. The signaling architecture of the model is developed for the sake of simplicity, and it may be argued that the simplifying assumptions render the model unrealistic. However, in most cases, computational modeling requires accounting for complex molecular details involved using certain simplifying assumptions. Some of the complex molecular details are DAG generation and lateral diffusion in the plasma membrane, precise stoichiometric binding of DAG with TRPC3, possibility of direct activation of TRPC3 by Ca^2+^ in the plasma membrane, and effects of cell-wide Ca^2+^ levels on the local TRPC3 signaling. Modeling all these complex molecular details is beyond the scope of this study. At its best, this study provides a mechanistic understanding by accounting for only the key signaling events involved in the assembly of TRPC3 signalosome in PCs.

For precision in PC, Ca^2+^ influx requires a high degree of specificity in TRPC3 signaling. This can be accomplished by tightly regulating the TRPC3 signaling in space and time. An interesting concept in cellular information processing system is the identification and existence of nodal hubs within a network where multiple signaling pathways converge to share information. It seems that chaperone proteins with adapting, scaffolding, and anchoring functions may assist in the assembly of these nodal hubs at discrete cellular locations by precisely regulating the localized pools of second messengers and kinase activity. These proteins modulate the integration and crosstalk of different signaling pathways through the formation of multimolecular complexes that incorporate the components of different pathways. These localized complexes are defined as signalosomes. These local signalosomes provide a high degree of specificity by integrating various upstream pathways and precisely controlling the downstream effectors. DAG, a critical and pleiotropic second messenger in PCs, may regulate multiple effectors. The question then arises how the specificity in DAG signaling can be achieved especially ensuring the precise amount of Ca^2+^ entry into PCs. Observations suggest that localized pools of DAG are regulated through the assembly of local machines as all the components of DAG synthesis, function, degradation, and even downstream effectors are found at the specific locations in the membrane of PCs. Through the dynamic organization of interlinked positive and negative feedback loops, the local TRPC3 signalosome in PCs confers tight control of DAG levels and Ca^2+^ influx in PCs.

## Conclusion

Here, based on the elementary interactions of key molecular players involved in the assembly of a local signalosome orchestrated by TRPC3 at parallel fiber–PC synapses, a mechanistic understanding of this signalosome is developed. This approach explains the key role of functional players involved in the TRPC3 signaling in PCs. This study shows how after purinergic receptor activation in PCs different molecular events may contribute to gating mechanism(s) leading to opening of TRPC3 and Ca^2+^ influx into PCs. This model, in part, can explain how DAG and Ca^2+^ homoeostasis might be regulated in PCs. The findings of this study may be useful in identifying the mechanistic basis of TRPC3 signaling and possible targeting opportunities of this channel protein in hereditary forms of human cerebellar ataxia.

## Data Availability

The original contributions presented in the study are included in the article/Supplementary Material, further inquiries can be directed to the corresponding author.
