# Supplementary material for: TRPC3 Channel Activity and Viability of Purkinje Neurons can be Regulated by a Local Signalosome
Source: Front Mol Biosci. 2022 Feb 21;9:818682. doi: 10.3389/fmolb.2022.818682 (PMC8899209; doi:10.3389/fmolb.2022.818682)
Supplement: Supplementary file 1 [file DataSheet1.pdf]

Supplementary Materials I: Supplementary Figures

Figure S_1: The three-compartment regulatory model of TRPC3 signaling in PCs. This model is based on full TRPC3 signaling in PCs. Here, the first compartment is extracellular space (0), the second compartment is the membrane (I), and the third compartment is cytosol (II). This model explains the spatial and temporal regulation of the DAG–TRPC3–PKCγ–DGKγ axis in PCs. The model also accounts for the calcium release through intracellular Ca^{2+} stores and influx through VDCC channels in the membrane compartment. This model describes a sequence of complex events involving the generation of second-messengers, translocation, activation, desensitization, and redistribution of DAG axis molecules, i.e., TRPC3, PKCγ, and DGKγ. This representation is specific to PCs. Here, the PKCγ molecule can be in four states: (1) dormant PKCγ residing in the cytosol ($PKC_{II}\gamma$); (2) active PKCγ residing in the cytosol ($PKC_{II}\gamma^{A}$); (3) inactive PKCγ residing in the membrane ($PKC_{I}\gamma$); (4) active PKCγ residing in the membrane ($PKC_{I}\gamma^{A}$). The other DAG effector kinase, i.e., DGKγ can be in three states: (1) dormant molecule residing in the cytosol ($DGK_{II}\gamma$); (2) inactive molecule residing in the membrane ($DGK_{I}\gamma$); (3) active and phosphorylated molecule residing in the membrane ($DGK_{I}\gamma_{P}$). The DAG effector channel TRPC3 can be in three states: (1) inactive channel residing in the membrane (TRPC3); (2) active channel molecule residing in the membrane ($TRPC3^{A}$); (3) phosphorylated and desensitized channel molecule residing in the membrane ($TRPC3_{P}^{A}$). Here, the VDCC molecule can be in two states: (1) inactive channel residing in membrane (VDCC) and active molecule residing in the membrane ($VDCC^{A}$). The VDCC channel is activated by the active form of TRPC3. Here, the channel molecules are only located in the membrane compartment and do not migrate to other compartments. Here, second-messenger DAG can either be in non-phosphorylated form (DAG) or in phosphorylated form (DAG_{P}). Second-messenger calcium can be either in the extracellular compartment (Ca_0^{2+}), membrane compartment (Ca_{I}^{2+}), or cytosolic compartment (Ca_{II}^{2+}). This three-compartment model explains that depolarization-induced activation of purinergic receptor leads to DAG generation in the membrane compartment, which in turn binds with channel TRPC3 and activates it, thus inducing calcium influx from the extracellular compartment to intracellular space. Additionally, it is also accounted for here that TRPC3 channel may permeate monovalent cations and can depolarize local dendrites which may activate VDCCs and induce Ca^{2+} influx. The release of Ca^{2+} from internal stores due GPCR mediated DAG/IP3 generation is also modeled here especially in the store version of this model. This calcium influx and release from internal stores stimulates the translocation of both PKCγ and DGKγ from the cytosolic compartment to the membrane. Once in the membrane compartment, PKCγ is activated through DAG binding; this active molecule, in turn, leads to the phosphorylation and activation of DGKγ in the membrane compartment. The active and phosphorylated DGKγ, in turn, induces the metabolism of DAG by catalyzing its phosphorylation. The active γ isoform of PKC also leads to negative feedback effect by phosphorylating and desensitizing the TRPC3 channel. This phosphorylation and desensitization event, in turn, leads to a positive feedback effect on the local DAG concentration, as it reduces the calcium influx from the extracellular compartment, thus reducing the cytosol-to-membrane translocation of both DAG effector molecules.

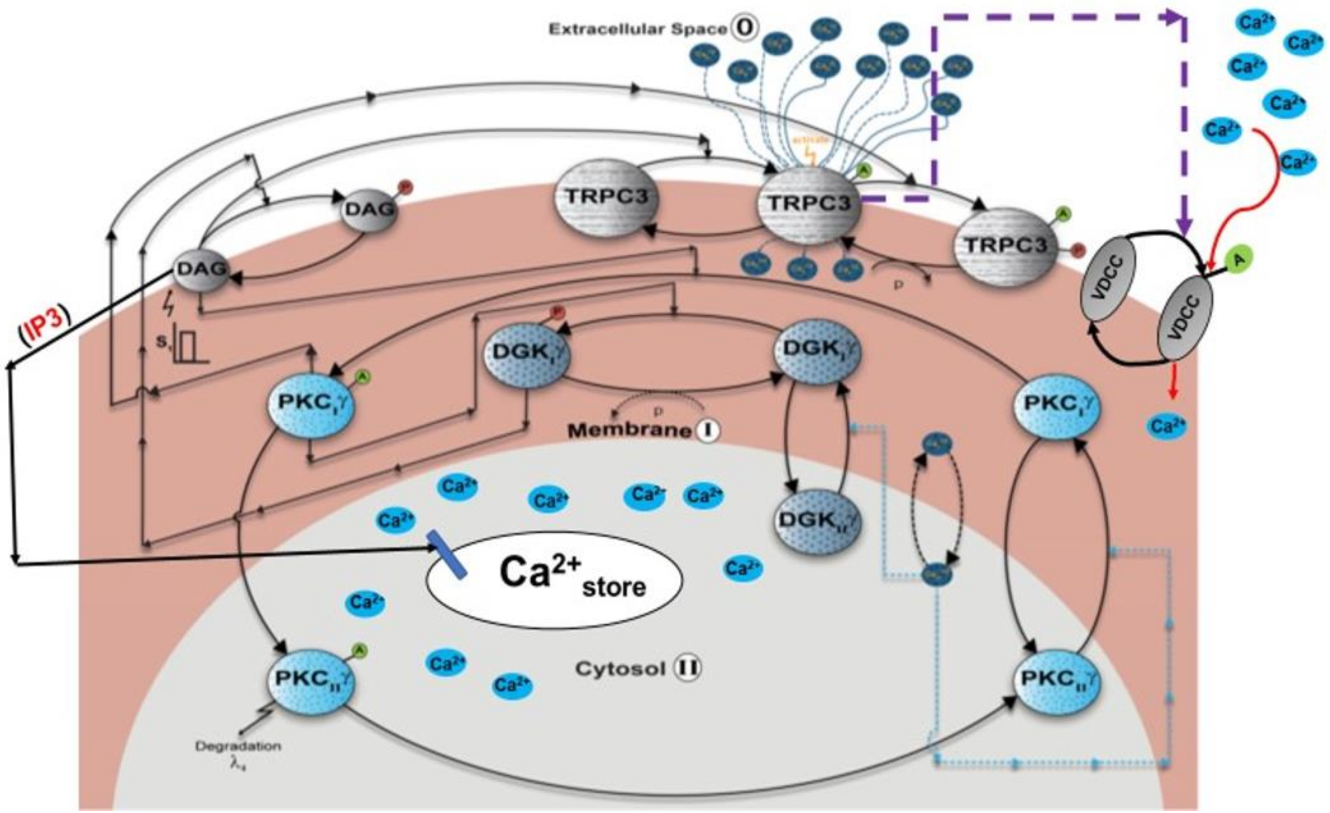

Extracellular Space O
TRPC3
TRPC3
TRPC3
DAG
DAG
(IP3)
DGK_I γ
DGK_I γ
DGK_II γ
PKC_I γ
PKC_I γ
PKC_II γ
PKC_II γ
VDCC
VDCC
Membrane I
Cytosol II
Ca2+ store
Degradation
Ca2+

Figure S_2: The simulations based on minimal TRPC3 model and mimicking the effect of blocking of, "k_6" (rate constant describing the phosphorylation of $DGK_I\gamma$ by $PKC_I\gamma$ at the membrane) on the translocation intensity and kinetics of PKCγ – DGKγ molecular pair during ATP-induced activation of purinergic receptor in CHO cells. (a) M/C ratio of PKCγ and DGKγ for no blocking case and 99.99 % blocking case of parameter k_6. (b) DAG temporal dynamics. (c) Ca^{+2} temporal dynamics in membrane compartment. (d) Ca^{+2} temporal dynamics in cytosolic compartment.

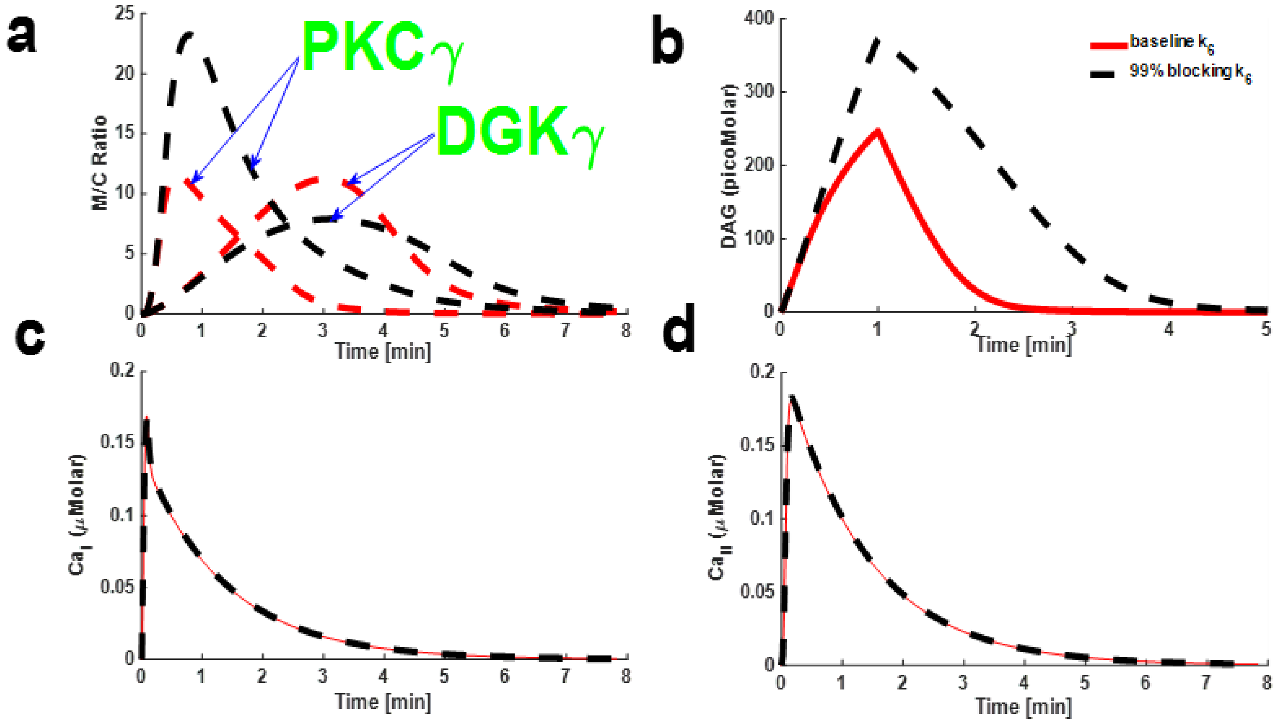

Figure S3: The simulations based on minimal TRPC3 model and mimicking the effect of enhancing k_{25} on translocation intensity and kinetics of PKCγ – DGKγ molecular pair and temporal dynamics of second messengers i.e., Ca^{2+} and DAG during ATP-induced activation of purinergic receptor in CHO cells. (a) M/C ratio of PKCγ and DGKγ for baseline case and 25-times increase of k_{25}. (b) temporal dynamics of second messenger DAG in membrane compartment. (c) Ca^{2+} temporal dynamics in membrane. (d) Ca^{2+} temporal dynamics in cytosolic compartment.

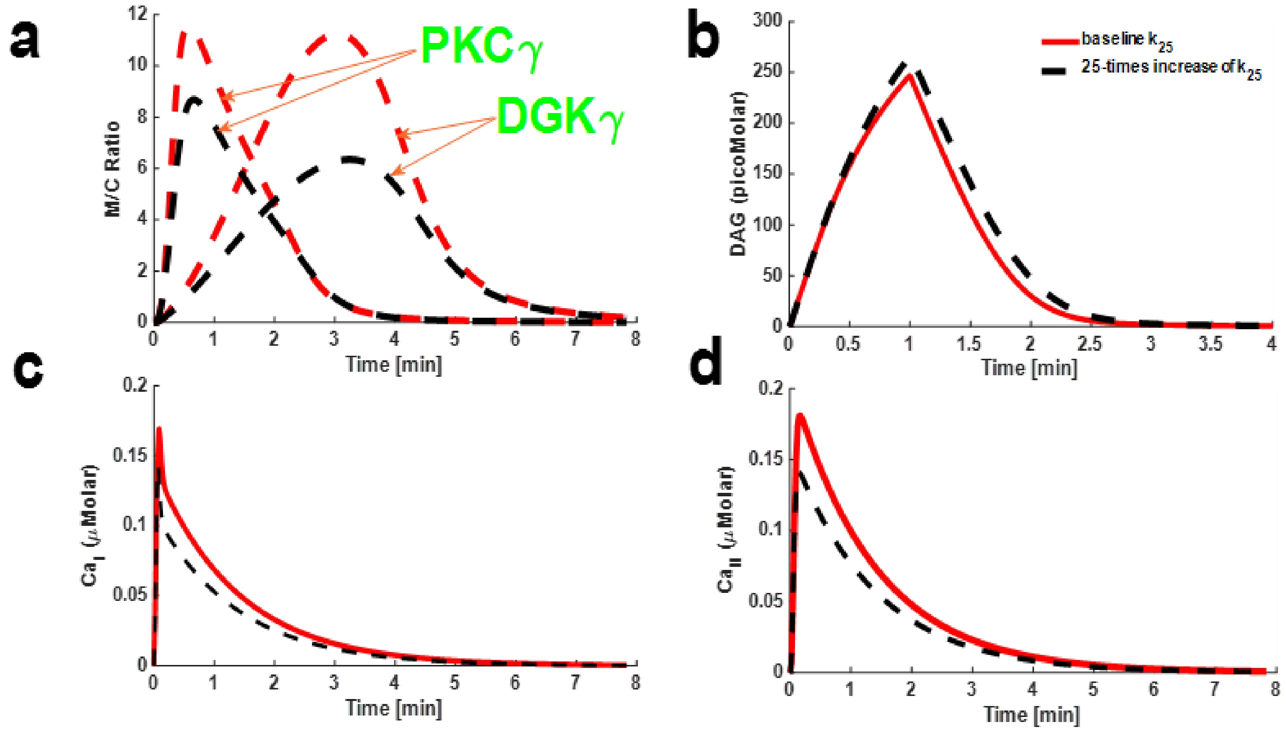

Figure S4: The simulations based on minimal TRPC3 model and mimicking the effect of TRPC3 expression on the translocation intensity and kinetics of PKCγ – DGKγ molecular pair during ATP-induced activation of purinergic receptor in CHO cells. (a) M/C ratio of PKCγ and DGKγ for baseline and a case of double than baseline TRPC3 expression. (b) temporal dynamics of second messenger DAG in membrane compartment. (c) Ca^{2+} temporal dynamics in membrane. (d) Ca^{2+} temporal dynamics in cytosol.

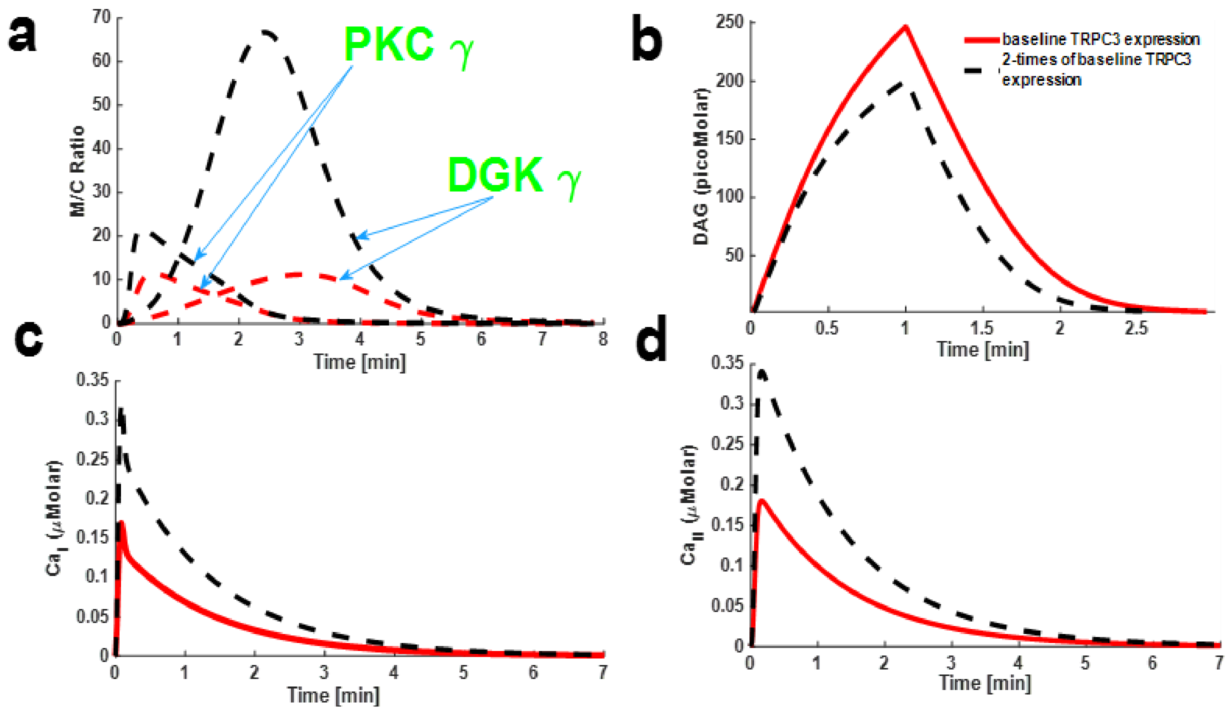

Figure S_5: The simulations based on minimal TRPC3 model and mimicking the effect of channel activation rate "k_{13}" on the TRPC3 signaling characteristics based on data from Purkinje cells. These results are based on store depletion version of model and without VDCCs. Thus, only Ca^{2+} contribution here is coming from TRPC3 mediated influx. These results show the translocation characteristics of the PKCγ and DGKγ molecular pair and dynamics of second messenger DAG and Ca^{+2} during KCl-induced purinergic receptor activation and stimulation of the TRPC3 signaling cascade in PCs. These results show that, in response to a brief 1-minute pulse, DAG is generated at the membrane, thus activating TRPC3 channel, which in turn allows the calcium flux into intracellular space and stimulates the translocation of PKCγ and DGKγ from cytosol to membrane. (a) M/C ratio of PKCγ at different blocking levels of k_{13} (b) M/C ratio of DGKγ at different blocking levels of k_{13}. (c) Ca^{+2} temporal dynamics in membrane compartment. (d) Ca^{+2} temporal dynamics in cytosolic compartment. (e) DAG temporal dynamics.

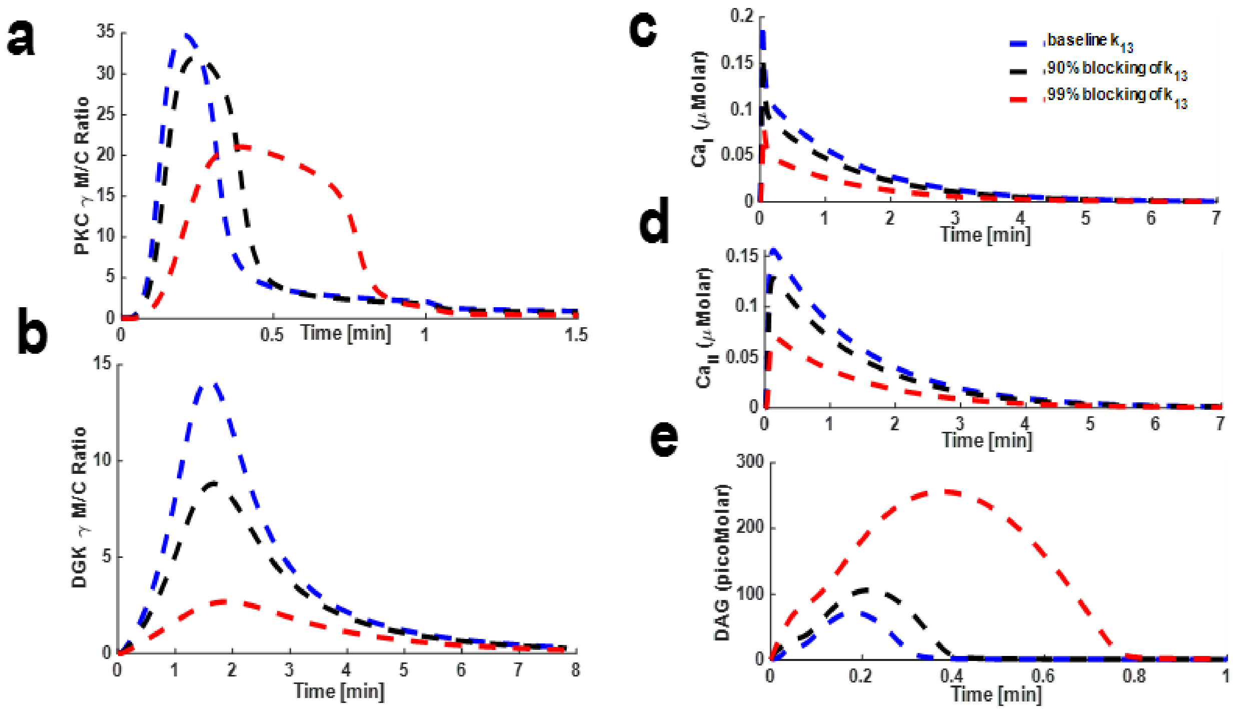

Figure S_6: Simulations based on minimal TRPC3 model and mimicking the effect of channel desensitization rate k_{25} on the TRPC3 signaling characteristics based on data from Purkinje cells. These results are based on store depletion version of model and without VDCCs. Thus, only Ca^{2+} contribution here is coming from TRPC3 mediated influx. These results show the translocation characteristics of the PKCγ and DGKγ molecular pair and dynamics of second messenger DAG and Ca^{2+} during KCl-induced purinergic receptor activation and stimulation of the TRPC3 signaling cascade in cPCs. These results show that, in response to a brief 1-minute pulse, DAG is generated at the membrane, thus activating TRPC3 channel, which in turn allows the calcium flux into intracellular space and stimulates the translocation of PKCγ and DGKγ from cytosol to membrane. (a) M/C ratio of PKCγ at different levels of k_{25} increase. (b) M/C ratio of DGKγ at different levels of k_{25} increase. (c) Ca^{2+} temporal dynamics in membrane compartment. (d) Ca^{2+} temporal dynamics in cytosolic compartment. (e) DAG temporal dynamics.

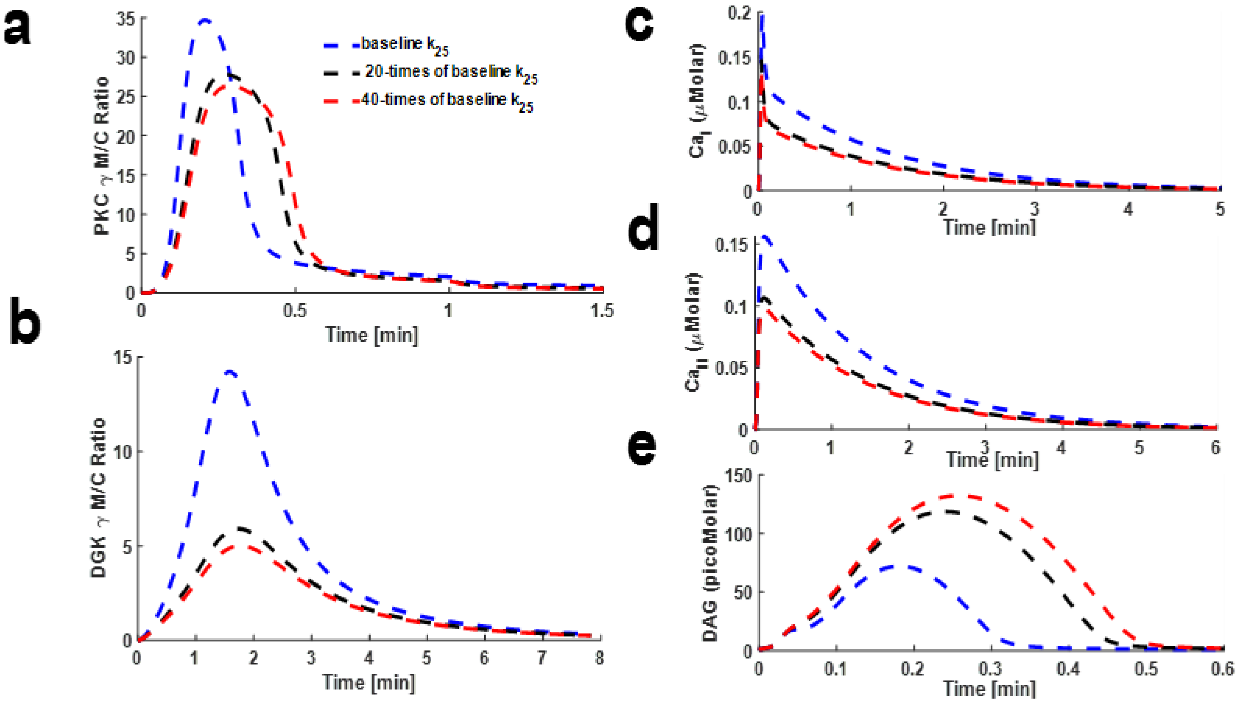

Figure S_7: The simulations based on full TRPC3 model and mimicking the effect of channel activation rate "k_{13}" on the TRPC3 signaling characteristics based on data from Purkinje cells. These results are based on cytosolic Ca^{2+} store version of model and with VDCCs in the membrane compartment. Thus, Ca^{2+} release and flux contributions are: 1.) release from internal stores; 2.) TRPC3 mediated influx; 3.) VDCCs mediated influx. These results show the translocation characteristics of the PKCγ and DGKγ molecular pair and dynamics of second messenger DAG and Ca^{2+} during KCl-induced purinergic receptor activation and stimulation of the TRPC3 signaling cascade in PCs. These results show that, in response to a brief 1-minute pulse, DAG is generated at the membrane, thus activating TRPC3 channel, which in turn allows the calcium flux into intracellular space and stimulates the translocation of PKCγ and DGKγ from cytosol to membrane. (a) M/C ratio of PKCγ at different blocking levels of k_{13} (b) M/C ratio of DGKγ at different blocking levels of k_{13}. (c) Ca^{2+} temporal dynamics in membrane compartment. (d) Ca^{2+} temporal dynamics in cytosolic compartment. (e) DAG temporal dynamics.

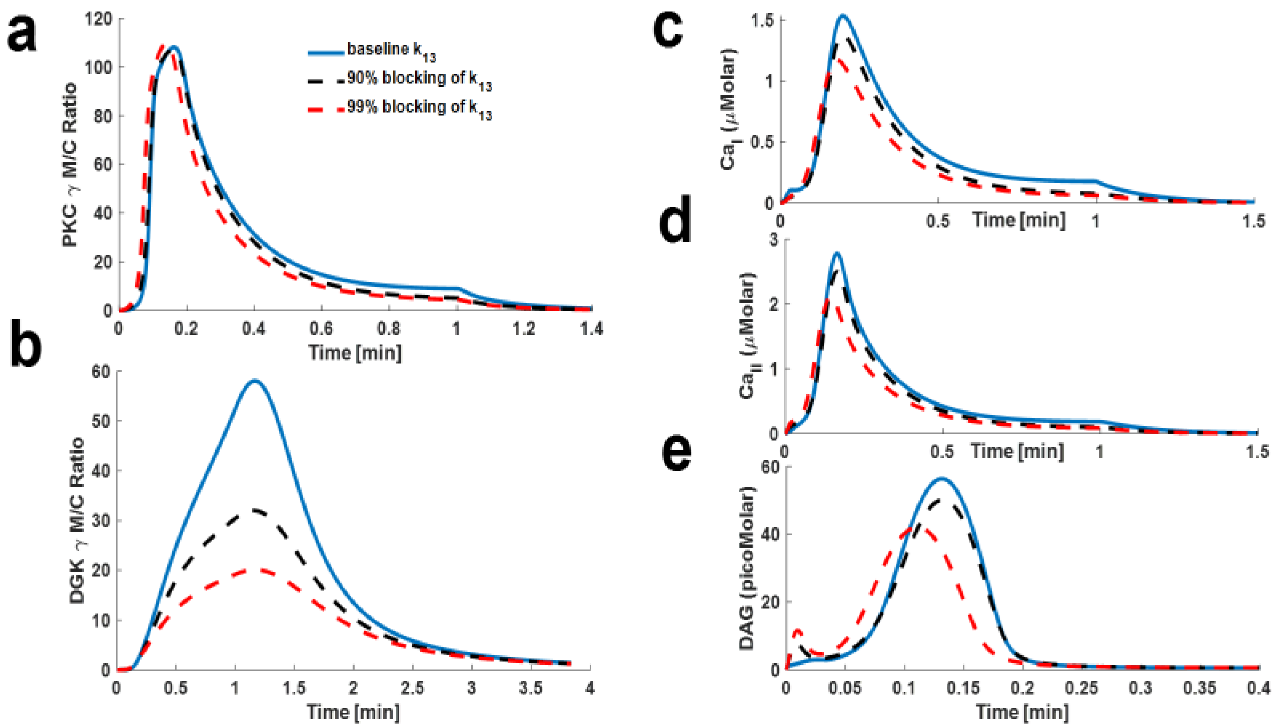

Figure S8: The simulations based on full TRPC3 model and mimicking the effect of blocking of, "k_6" (rate constant describing the phosphorylation of $DGK_I\gamma$ by $PKC_I\gamma$ at the membrane) on the translocation intensity and kinetics of PKCγ – DGKγ molecular pair. These results are based on cytosolic Ca^{2+} store version of model and with VDCCs in the membrane compartment. Thus, Ca^{2+} release and flux contributions are: 1.) release from internal stores; 2.) TRPC3 mediated influx; 3.) VDCCs mediated influx. These results show the translocation characteristics of the PKCγ and DGKγ molecular pair and dynamics of second messenger DAG and Ca^{2+} during KCl-induced purinergic receptor activation and stimulation of the TRPC3 signaling cascade in PCs. These results show that, in response to a brief 1-minute pulse, DAG is generated at the membrane, thus activating TRPC3 channel, which in turn allows the calcium flux into intracellular space and stimulates the translocation of PKCγ and DGKγ from cytosol to membrane. (a) M/C ratio of PKCγ at different blocking levels of k_{13} (b) M/C ratio of DGKγ at different blocking levels of k_{13}. (c) Ca^{2+} temporal dynamics in membrane compartment. (d) Ca^{2+} temporal dynamics in cytosolic compartment. (e) DAG temporal dynamics.

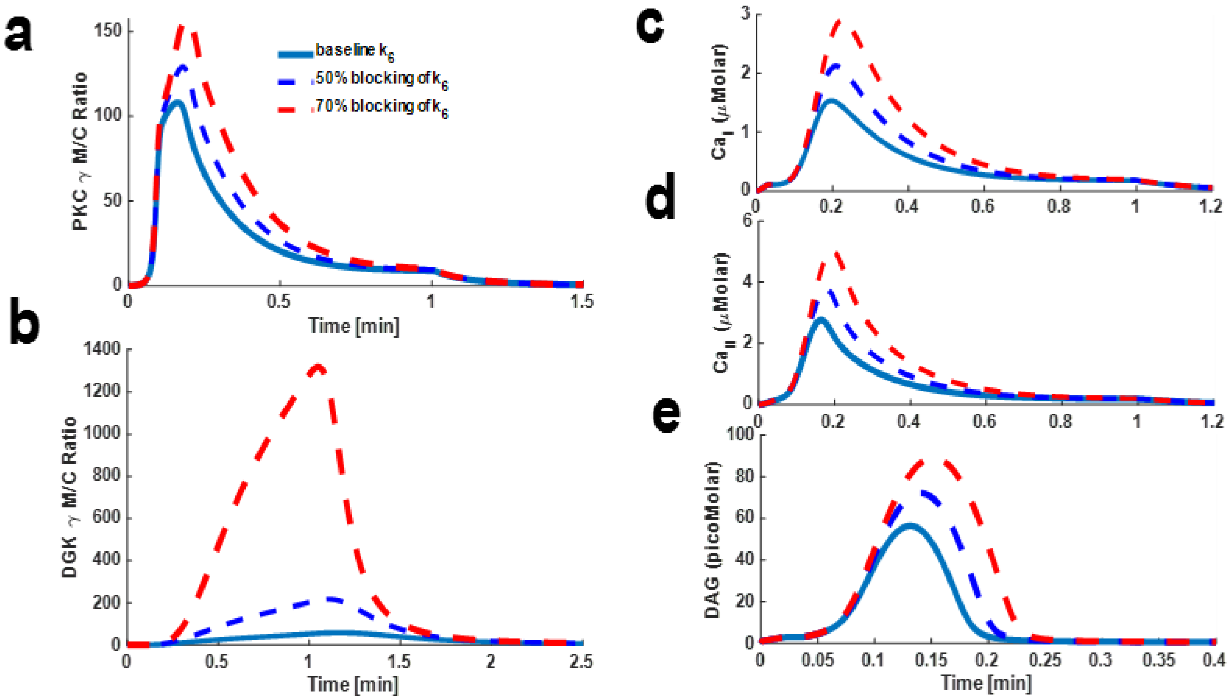

Figure S9: Simulations comparing the effects of alteration of the TRPC3 expression with full channel activity case with a case of TRPC3 expression variation and 99% blocking of channel activity based on data from Purkinje cells. These results are based on full model of TRPC3 signaling which along with TRPC3-mediated influx also accounts for cytosolic release of Ca^{2+} from stores and VDCCs-mediated influx in the membrane compartment. Thus, Ca^{2+} release and flux contributions are: 1.) release from internal stores; 2.) TRPC3 mediated influx; 3.) VDCCs mediated influx. These results show the translocation characteristics of the PKCγ and DGKγ molecular pair and dynamics of second messenger DAG and Ca^{2+} during KCl-induced purinergic receptor activation and stimulation of the TRPC3 signaling cascade in PCs. These results show that, in response to a brief 1-minute pulse, DAG is generated at the membrane, thus activating TRPC3 channel, which in turn allows the calcium flux into intracellular space and stimulates the translocation of PKCγ and DGKγ from cytosol to membrane. (a) M/C ratio of PKCγ. (b) M/C ratio of DGKγ. (c) Ca^{2+} temporal dynamics in membrane compartment. (d) Ca^{2+} temporal dynamics in cytosolic compartment. (e) DAG temporal dynamics.

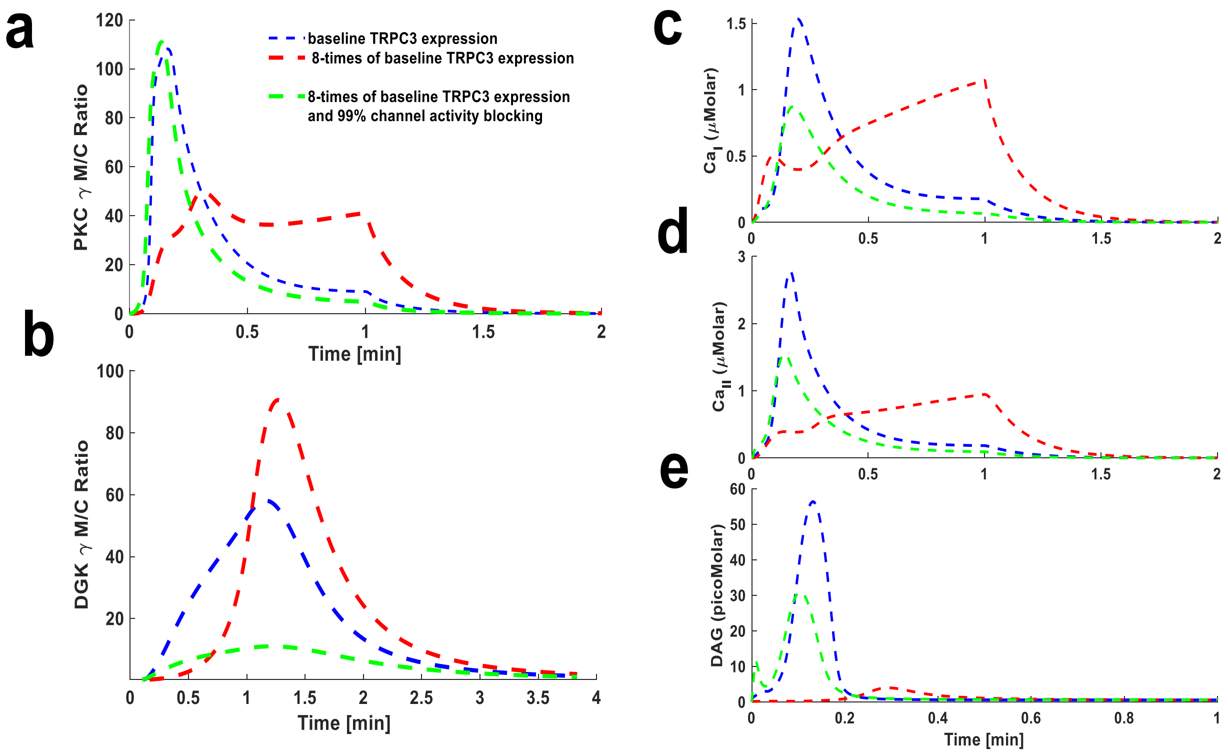

Figure S_{10}: Simulations comparing the case of no constitutive activity of basal state of TRPC3 with the case of constitutively active TRPC3 even in its basal state. These simulations are based on minimal TRPC3 model and mimicking the effect of constitutive TRPC3 activity on the signaling characteristics of TRPC3 signalosome based on data from Purkinje cells. These results are based on store depletion version of model and without VDCCs. Thus, only Ca^{2+} contribution here is coming from TRPC3 mediated influx. These results show the translocation characteristics of the PKCγ and DGKγ molecular pair and dynamics of second messenger DAG and Ca^{2+} during KCl-induced purinergic receptor activation and stimulation of the TRPC3 signaling cascade in cPCs. These results show that, in response to a brief 1-minute pulse, DAG is generated at the membrane, thus activating TRPC3 channel, which in turn allows the calcium flux into intracellular space and stimulates the translocation of PKCγ and DGKγ from cytosol to membrane. (a) M/C ratio of PKCγ at different levels of k_{25} increase. (b) M/C ratio of DGKγ at different levels of k_{25} increase. (c) Ca^{2+} temporal dynamics in membrane compartment. (d) Ca^{2+} temporal dynamics in cytosolic compartment. (e) DAG temporal dynamics.

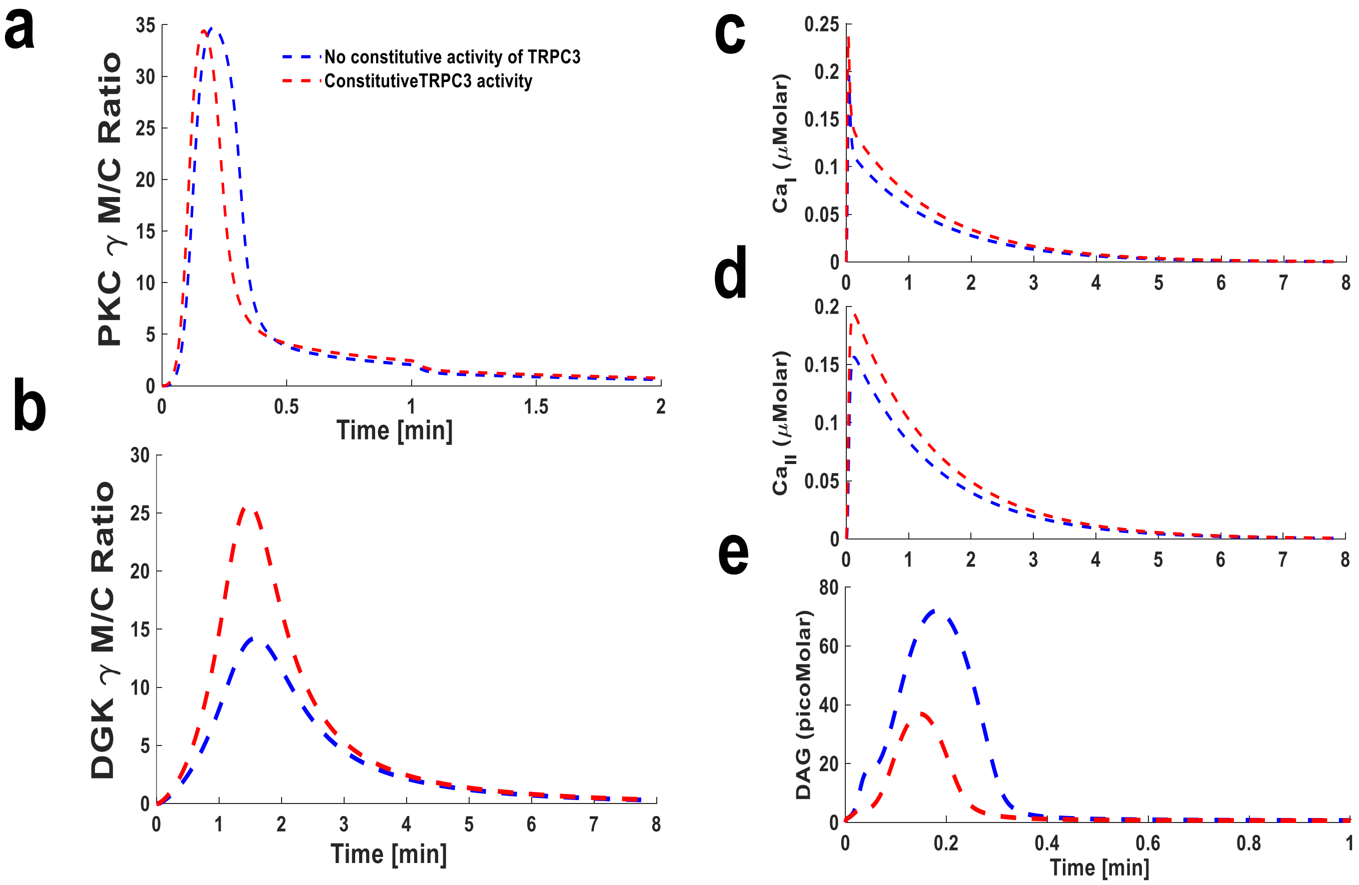

Supplementary Material 2: Table 1: Numerical values of biochemical rate parameters for local TRPC3 Signalosome in CHO cells as described in the (a) minimal model of Materials and Methods in main manuscript and biochemical reaction equations1-22.

**Parameter**	**Description**	**Numerical Values**
k_1	Kinetic rate constant for DAG generation	1.0 sec^{-1}
k_2	Association rate constant $PKC\gamma_I$ - DAG binding.	0.95 $picoM^{-1}sec^{-1}$
k_3	Dissociation rate constant $PKC\gamma_I^A$	1.0 sec^{-1}
k_4	Association rate constant $PKC\gamma_I^A$ - $DGK_I\gamma$ binding.	0.98 $picoM^{-1}sec^{-1}$
k_5	Dissociation rate constant C_1	0.05 sec^{-1}
k_6	Rate constant for the phosphorylation of $DGK_I\gamma$	0.98 sec^{-1}
k_7	Rate constant of $DGK_I\gamma_P$ de-phosphorylation	0.5 sec^{-1}
k_8	Association rate constant $DGK_I\gamma_P$ and DAG	0.92 $picoM^{-1}sec^{-1}$
k_9	Dissociation rate constant for C_2	100 sec^{-1}
k_{10}	Rate Constant of DAG phosphorylation	0.91 sec^{-1}
k_{11}	Rate constant of DAG_P conversion to P.A	0.2 sec^{-1}
k_{12}	Rate constant of dephosphorylation of DAG_P	0.001 sec^{-1}
k_{13}	Association rate constant TRPC3 and DAG	0.65 $picoM^{-1}sec^{-1}$
k_{14}	Dissociation rate constant $TRPC3^A$	0.185 sec^{-1}
k_{15}	Rate constant for Ca^{+2} influx into cellular space due to TRPC3 gating	0.0018*$TRPC3^A$ sec^{-1}
k_{16}	Rate constant for Ca^{+2} outflux	0.03 sec^{-1}
k_{17}	Ca^{+2} translocation rate from membrane-to-cytosol	0.398 sec^{-1}
k_{18}	Ca^{+2} re-translocation rate from cytosol-to-membrane	0.287 sec^{-1}
k_{19}	Ca^{+2} binding with $PKC\gamma$ in cytosol	0.78 $picoM^{-1}sec^{-1}$
k_{20}	Dissociation of $PKC\gamma$*	0.186 sec^{-1}
k_{21}	Ca^{+2} binding with $DGK\gamma$ in cytosol	0.078 $picoM^{-1}sec^{-1}$

k_{22}	Dissociation of DGKγ*	0.0186 sec^{-1}
k_{23}	Association rate constant of $TRPC3^{A}$ and $PKC_{I}\gamma^{A}$	0.987 $picoM^{-1}sec^{-1}$
k_{24}	Dissociation of complex C_3	0.125 sec^{-1}
k_{25}	Phosphorylation of $TRPC3^{A}$	0.657 sec^{-1}
k_{26}	Dephosphorylation of $TRPC3^{A}_{P}$	0.015 sec^{-1}
ko	Rate constant for the deactivation of $PKC\gamma_{II}^{A}$	1.0 sec^{-1}
λ_0	Translocation rate of $PKC_{II}\gamma$* from cytosol to plasma membrane	0.000001*Ca_{II} sec^{-1}
λ_{00}	Re-translocation rate of $PKC_{I}\gamma$ from plasma membrane to cytosol	0.1 sec^{-1}
λ_5	Translocation rate of $DGK_{II}\gamma$* from cytosol to plasma membrane	0.00000019*CaII sec^{-1}
λ_{55}	Re-translocation rate of $DGK_{I}\gamma$ from plasma membrane to cytosol	0.1 sec^{-1}
λ_3	Re-translocation rate of $PKC_{I}\gamma^{A}$ from plasma membrane to cytosol	0.01 sec^{-1}
λ_4	Degradation rate of $PKC_{II}\gamma^{A}$	0.0005 sec^{-1}
S_1	Strength of DAG pulse	7 pico-Molar
Ca^{+2}Ext	Strength of extracellular Ca^{+2} pulse	1.2 *10000000 pico-Molar
P	Phosphatase concentration	0.02 pico-Molar

Supplementary Material 3: Table 2: Numerical values of biochemical rate parameters for the fast kinetics local TRPC3 Signalosome in wild-type Purkinje cells of the cerebellum as described in the (a) minimal model of Materials and Methods in main manuscript and biochemical reaction equations1-22.

**Parameter**	**Description**	**Numerical Values**
k_1	Kinetic rate constant for DAG generation	1.0 sec^{-1}
k_2	Association rate constant $PKC\gamma_I$ - DAG binding.	0.95 $picoM^{-1}sec^{-1}$
k_3	Dissociation rate constant $PKC\gamma_I^A$	1.0 sec^{-1}
k_4	Association rate constant $PKC\gamma_I^A$ - $DGK_I\gamma$ binding.	0.995 $picoM^{-1}sec^{-1}$
k_5	Dissociation rate constant C_1	0.05 sec^{-1}
k_6	Rate constant for the phosphorylation of $DGK_I\gamma$	0.899 sec^{-1}
k_7	Rate constant of $DGK_I\gamma_P$ de-phosphorylation	0.5 sec^{-1}
k_8	Association rate constant $DGK_I\gamma_P$ and DAG	0.95 $picoM^{-1}sec^{-1}$
k_9	Dissociation rate constant for C_2	0.1 sec^{-1}
k_{10}	Rate Constant of DAG phosphorylation	0.99 sec^{-1}
k_{11}	Rate constant of DAG_P conversion to P.A	0.1 sec^{-1}
k_{12}	Rate constant of dephosphorylation of DAG_P	0.1 sec^{-1}
k_{13}	Association rate constant TRPC3 and DAG	0.65 $picoM^{-1}sec^{-1}$
k_{14}	Dissociation rate constant $TRPC3^A$	0.185 sec^{-1}
k_{15}	Rate constant for Ca^{+2} influx into cellular space due to TRPC3 gating	0.00125*$TRPC3^A$ sec^{-1}
k_{16}	Rate constant for Ca^{+2} outflux	0.03 sec^{-1}
k_{17}	Ca^{+2} translocation rate from membrane-to-cytosol	0.398 sec^{-1}
k_{18}	Ca^{+2} re-translocation rate from cytosol-to-membrane	0.287 sec^{-1}
k_{19}	Ca^{+2} binding with $PKC\gamma$ in cytosol	0.78 $picoM^{-1}sec^{-1}$
k_{20}	Dissociation of $PKC\gamma^*$	0.186 sec^{-1}
k_{21}	Ca^{+2} binding with $DGK\gamma$ in cytosol	0.078 $picoM^{-1}sec^{-1}$
k_{22}	Dissociation of $DGK\gamma^*$	0.0186 sec^{-1}
k_{23}	Association rate constant of $TRPC3^A$ and $PKC_I\gamma^A$	0.987 $picoM^{-1}sec^{-1}$

k_{24}	Dissociation of complex C_3	0.125 sec^{-1}
k_{25}	Phosphorylation of $TRPC3^A$	0.657 sec^{-1}
k_{26}	Dephosphorylation of $TRPC3^A{}_P$	0.015 sec^{-1}
ko	Rate constant for the deactivation of $PKC\gamma_{II}{}^A$	1.0 sec^{-1}
λ_0	Translocation rate of $PKC_{II}\gamma^*$ from cytosol to plasma membrane	$0.00000498*Ca_{II}$ sec^{-1}
λ_{00}	Re-translocation rate of $PKC_I\gamma$ from plasma membrane to cytosol	0.34 sec^{-1}
λ_5	Translocation rate of $DGK_{II}\gamma^*$ from cytosol to plasma membrane	$0.00000032*Ca_{II}$ sec^{-1}
λ_{55}	Re-translocation rate of $DGK_I\gamma$ from plasma membrane to cytosol	0.01 sec^{-1}
λ_3	Re-translocation rate of $PKC_I\gamma^A$ from plasma membrane to cytosol	0.01 sec^{-1}
λ_4	Degradation rate of $PKC_{II}\gamma^A$	0.0005 sec^{-1}
S_1	Strength of DAG pulse	32 pico-Molar
$Ca^{2+}Ext$	Strength of extracellular Ca^{+2} pulse	4.0*1000000 pico-Molar
P	Phosphatase concentration	0.02 pico-Molar

Supplementary Material 4: Table 3: Numerical values of biochemical rate parameters for the fast kinetics local TRPC3 Signalosome in wild-type Purkinje cells of the cerebellum as described in the (a) minimal model of Materials and Methods in main manuscript and biochemical reaction equations1-22 and equations 23-25.

**Parameter**	**Description**	**Numerical Values**
k_1	Kinetic rate constant for DAG generation	1.0 sec^{-1}
k_2	Association rate constant $PKC\gamma_I$ - DAG binding.	0.95 $picoM^{-1}sec^{-1}$
k_3	Dissociation rate constant $PKC\gamma_I^A$	1.0 sec^{-1}
k_4	Association rate constant $PKC\gamma_I^A$ - $DGK_I\gamma$ binding.	0.995 $picoM^{-1}sec^{-1}$
k_5	Dissociation rate constant C_1	0.05 sec^{-1}
k_6	Rate constant for the phosphorylation of $DGK_I\gamma$	0.899 sec^{-1}
k_7	Rate constant of $DGK_I\gamma_P$ de-phosphorylation	0.5 sec^{-1}
k_8	Association rate constant $DGK_I\gamma_P$ and DAG	0.95 $picoM^{-1}sec^{-1}$
k_9	Dissociation rate constant for C_2	0.1 sec^{-1}
k_{10}	Rate Constant of DAG phosphorylation	0.99 sec^{-1}
k_{11}	Rate constant of DAG_P conversion to P.A	0.1 sec^{-1}
k_{12}	Rate constant of dephosphorylation of DAG_P	0.1 sec^{-1}
k_{13}	Association rate constant TRPC3 and DAG	0.65 $picoM^{-1}sec^{-1}$
k_{14}	Dissociation rate constant $TRPC3^A$	0.185 sec^{-1}
k_{15}	Rate constant for Ca^{+2} influx into cellular space due to TRPC3 gating	0.00125*$TRPC3^A$ sec^{-1}
k_{16}	Rate constant for Ca^{+2} outflux	0.03 sec^{-1}
k_{17}	Ca^{+2} translocation rate from membrane-to-cytosol	0.398 sec^{-1}
k_{18}	Ca^{+2} re-translocation rate from cytosol-to-membrane	0.287 sec^{-1}
k_{19}	Ca^{+2} binding with $PKC\gamma$ in cytosol	0.78 $picoM^{-1}sec^{-1}$
k_{20}	Dissociation of $PKC\gamma^*$	0.186 sec^{-1}
k_{21}	Ca^{+2} binding with $DGK\gamma$ in cytosol	0.078 $picoM^{-1}sec^{-1}$
k_{22}	Dissociation of $DGK\gamma^*$	0.0186 sec^{-1}

k_{23}	Association rate constant of $TRPC3^A$ and $PKC_I\gamma^A$	0.987 $picoM^{-1}sec^{-1}$
k_{24}	Dissociation of complex C_3	0.125 sec^{-1}
k_{25}	Phosphorylation of $TRPC3^A$	0.657 sec^{-1}
k_{26}	Dephosphorylation of $TRPC3^A{}_P$	0.015 sec^{-1}
ko	Rate constant for the deactivation of $PKC\gamma_{II}{}^A$	1.0 sec^{-1}
λ_0	Translocation rate of $PKC_{II}\gamma^*$ from cytosol to plasma membrane	$0.00000498*Ca_{II}$ sec^{-1}
λ_{00}	Re-translocation rate of $PKC_I\gamma$ from plasma membrane to cytosol	0.15 sec^{-1}
λ_5	Translocation rate of $DGK_{II}\gamma^*$ from cytosol to plasma membrane	$0.0000001*Ca_{II}$ sec^{-1}
λ_{55}	Re-translocation rate of $DGK_I\gamma$ from plasma membrane to cytosol	0.01 sec^{-1}
λ_3	Re-translocation rate of $PKC_I\gamma^A$ from plasma membrane to cytosol	0.01 sec^{-1}
λ_4	Degradation rate of $PKC_{II}\gamma^A$	0.0005 sec^{-1}
S_1	Strength of DAG pulse	32 pico-Molar
Ca^{2+}Ext	Strength of extracellular Ca^{2+} pulse	4.0*1000000 pico-Molar
P	Phosphatase concentration	0.02 pico-Molar
VDCC	Expression of VDCC	2 pico-molar
Ca^{2+}Stores	Concentration in the stores	8.0*1000000 pico-molar
k_{27}	Rate of VDCC and $TRPC3^A$ complex formation	0.185 $picoM^{-1}sec^{-1}$
k_{28}	Dissociation of complex C_4	0.01 sec^{-1}
k_{29}	Rate of activation of VDCC channel	0.0235 sec^{-1}
k_{31}	Rate constant for Ca^{2+} outflux	0.0027 sec^{-1}
k_{33}	Rate constant for Ca^{2+} flux back to stores	0.17 sec^{-1}
k_{30}	Rate constant for Ca^{2+} influx into cellular space due to VDCC channel	$0.001*VDCC^A$ sec^{-1}
k_{32}	Rate constant for Ca^{2+} release into cytosolic space.	0.00285* DAG sec^{-1}

Supplementary Material 5: Differential equations describing the minimal model of local TRPC3 Signalosome (Figures:1, 2 and 3). These are based on standard mass action kinetics applied to elementary biochemical reactions as described in reactions R1 to R22.

1. **$PKC_{II}\gamma$ in the second compartment: Variable # 1**

d[**γPKC_{II}**]/dt = -k19*CaII.*rPKCII + k20*rPKCIIACTIVESTAR + lambda00*rPKCI + k0*rPKCIIACTIVE -------- **[1]**

2. **$PKC_{I}\gamma$ in the first compartment: Variable # 2**

d[**$rPKC_{I}$**] /dt = lambda0.*rPKCIIACTIVESTAR - lambda00.*rPKCI - k2.*DAG.*rPKCI + k3.*rPKCIACTIVE -------- **[2]**

3. **$DGK_{II}\gamma$ in the second compartment: Variable # 3**

d[**DGKIIr**]/dt = lambda55.*DGKIr - k21.*DGKIIr.*CaII + k22.*DGKIIrSTAR------ **[3]**

4. **$DGK_{I}\gamma$ in the first compartment: Variable # 4**

d[**DGKIr**]/dt = lambda5.*DGKIIrSTAR - lambda55.*DGKIr - k4.*rPKCIACTIVE.*DGKIr + k5.*C1 + k7.*DGKIrP.*P ------------------**[4]**

5. **DAG: Variable # 5**

d[**DAG**]/dt = k1.*S1 - k2.*rPKCI.*DAG + k3.*rPKCIACTIVE - k8.*DGKIrP.*DAG + k9.*C2 + k0.*rPKCIIACTIVE + k12.*DAGP.*P - lambda1.*DAG - k13.*TRPC3.*DAG + k14.*TRPC3A ------------**[5]**

6. **DAG_{P}: Variable # 6**

d[**DAG_{P}**]/dt = -k12.*DAGP.*P - k11.*DAGP - lambda2.*DAGP + k10.*C2 -------- **[6]**

7. **$PKC_{I}r^{active}$ in the first compartment: Variable # 7**

d[**$PKC_{I}r$**]/dt = k2.*DAG.*rPKCI - k3.*rPKCIACTIVE - k4.*rPKCIACTIVE.*DGKIr + k5.*C1 + k6.*C1 - lambda3.*rPKCIACTIVE - k23.*rPKCIACTIVE.*TRPC3A + k24.*C3 + k25.*C3 ------------ **[7]**

8. **DGKIrP in the first compartment: Variable # 8**

d[**$DGK_I rP$**]/dt = -k7.*DGKIrP.*P + k6.*C1 - k8.*DGKIrP.*DAG + k9.*C2 + k10.*C2 **-------------------[8]**

9. **C_1: Variable # 9**

d[**C_1**]/dt = k4.*rPKCIACTIVE.*DGKIr - k5.*C1 - k6.*C1 **--------- [9]**

10. **C_2: Variable # 10**

d[**C_2**]/dt = k8.*DGKIrP.*DAG - k9.*C2 - k10.*C2 **---------[10]**

11. **rPKCIIACTIVE: active PKCgamma in second compartment: Variable #11**

d [**rPKCIIACTIVE**] /dt = lambda3.*rPKCIACTIVE - lambda4.*rPKCIIACTIVE - k0.*rPKCIIACTIVE **--------------- [11]**

12. **rPKCIIACTIVESTAR: Variable # 12**

d[**rPKCIIACTIVESTAR**]/dt = -lambda0.*rPKCIIACTIVESTAR + k19.*rPKCII.*CaII - k20.*rPKCIIACTIVESTAR **----------------- [12]**

13. **DGKIIrSTAR: Variable # 13**

d **[DGKIIrSTAR**] /dt13 = -lambda5.*DGKIIrSTAR + k21.*CaII.*DGKIIr -k22.*DGKIIrSTAR **---------------- [13]**

14. **Ca^{2+}_I: Variable # 14**

d [**Ca^{2+}_I**]/dt14 = k15.*CaExternal - k16.*CaI - k17.*CaI + k18.*CaII + lambda00.*rPKCI + lambda55.*DGKIr **-------------------[14]**

15. Ca^{2+}_{II}: Variable # 15

d **[Ca^{2+}_{II}]/** dt15 = k17.*CaI - k18.*CaII - k19.*rPKCII.*CaII + k20.*rPKCIIACTIVESTAR - k21.*DGKIIr.*CaII + k22.*DGKIIrSTAR -------------- **[15]**

16. C13 : Variable # 16

d[**C3**]/dt = k23.*rPKCIACTIVE.*TRPC3A -k24.*C3 - k25.*C3 --------- **[16]**

17. TRPC3: Variable # 17

d[**TRPC3**]/dt = -k13.*TRPC3.*DAG + k14.*TRPC3A ---------- **[17]**

18. $TRPC3^{A}$: Variable # 18

d[**$TRPC3^{A}$**]/dt = k13.*TRPC3.*DAG - k14.*TRPC3A -k23.*rPKCIACTIVE.*TRPC3A + k24.*C3 + k26.*TRPC3AP.*P ------------- **[18]**

19. $TRPC3^{A}_{P}$: Variable # 19

d[**$TRPC3^{A}_{P}$**]/dt = k25.*C3 -k26.*TRPC3AP.*P ------------ **[19]**
